# An O Antigen Capsule Modulates Bacterial Pathogenesis in *Shigella sonnei*

**DOI:** 10.1371/journal.ppat.1004749

**Published:** 2015-03-20

**Authors:** Mariaelena Caboni, Thierry Pédron, Omar Rossi, David Goulding, Derek Pickard, Francesco Citiulo, Calman A. MacLennan, Gordon Dougan, Nicholas R. Thomson, Allan Saul, Philippe J. Sansonetti, Christiane Gerke

**Affiliations:** 1 Novartis Vaccines Institute for Global Health, Siena, Via Fiorentina, Italy; 2 Institut Pasteur, Unité de Pathogénie Microbienne Moléculaire, INSERM U1202, Paris, France; 3 Wellcome Trust Sanger Institute, Hinxton, Cambridgeshire, United Kingdom; 4 Collège de France, Chaire de Microbiologie et Maladies Infectieuses, Paris, France; The University of Texas-Houston Medical School, UNITED STATES

## Abstract

*Shigella* is the leading cause for dysentery worldwide. Together with several virulence factors employed for invasion, the presence and length of the O antigen (OAg) of the lipopolysaccharide (LPS) plays a key role in pathogenesis. *S*. *flexneri* 2a has a bimodal OAg chain length distribution regulated in a growth-dependent manner, whereas *S*. *sonnei* LPS comprises a monomodal OAg. Here we reveal that *S*. *sonnei*, but not *S*. *flexneri* 2a, possesses a high molecular weight, immunogenic group 4 capsule, characterized by structural similarity to LPS OAg. We found that a *galU* mutant of *S*. *sonnei*, that is unable to produce a complete LPS with OAg attached, can still assemble OAg material on the cell surface, but a *galU* mutant of *S*. *flexneri* 2a cannot. High molecular weight material not linked to the LPS was purified from *S*. *sonnei* and confirmed by NMR to contain the specific sugars of the *S*. *sonnei* OAg. Deletion of genes homologous to the group 4 capsule synthesis cluster, previously described in *Escherichia coli*, abolished the generation of the high molecular weight OAg material. This OAg capsule strongly affects the virulence of *S*. *sonnei*. Uncapsulated knockout bacteria were highly invasive *in vitro* and strongly inflammatory in the rabbit intestine. But, the lack of capsule reduced the ability of *S*. *sonnei* to resist complement-mediated killing and to spread from the gut to peripheral organs. In contrast, overexpression of the capsule decreased invasiveness *in vitro* and inflammation *in vivo* compared to the wild type. In conclusion, the data indicate that in *S*. *sonnei* expression of the capsule modulates bacterial pathogenesis resulting in balanced capabilities to invade and persist in the host environment.

## Author Summary

Shigellosis is a major global health concern. Recently, a shift in the dominance of types of *Shigella* that cause disease has been observed with *S*. *sonnei* increasing in prevalence under improved socio-economic conditions leading to a replacement of *S*. *flexneri*. Most of the knowledge of *Shigella* disease mechanisms has been obtained from studies of *S*. *flexneri*. We found that *S*. *sonnei* possesses a high molecular weight sugar capsule that is absent in *S*. *flexneri* 2a. Removal of the capsule made *S*. *sonnei* bacteria highly invasive *in vitro* and strongly inflammatory *in vivo*, but in contrast, there was reduced spreading of these mutant bacteria from the gut to peripheral organs in rabbits and higher sensitivity to complement-mediated lysis. Thus, the capsule plays a role in both, invasion and protection of the bacteria against the innate immune defense of the host during the infection. These findings indicate that the capsule is an important virulence factor for *S*. *sonnei*. Based on the substantial changes in pathogenesis observed upon removal and overexpression of the capsule, we hypothesize that its level of expression may be under significant evolutionary pressure.

## Introduction

Shigellosis, or bacillary dysentery, is an acute human inflammatory disease of the large intestine, characterized by watery diarrhea, fever, abdominal pain, and bloody and mucus stools, caused by Gram-negative *Shigella* enterobacteria [1]. This disease is a major global health concern, responsible for more than 7 million Disability-Adjusted Life Years and 100,000 deaths per year [2], predominantly affecting children under 5 years of age from developing countries [3]. Deaths caused by shigellosis have been linked to intestinal but also systemic complications, including pneumonia, hypoglycemia, and hemolytic-uremic syndrome [4]. *Shigella* bacteremia is generally rare in adults and in individuals with no underlying condition but has been described in young children with frequencies up to 7% of cases [5,6], with malnutrition being an important risk factor, or in immunocompromised individuals [7] and has been associated with high mortality rates [5,6,7,8].

Fifty *Shigella* serotypes belonging to the four serogroups of the genus (*S*. *dysenteriae*, *S*. *flexneri*, *S*. *boydii*, *S*. *sonnei*) are distinguished based on the structure of the O antigen (OAg) polysaccharide of the lipopolysaccharide (LPS) [9]. Among them, *S*. *flexneri* and *S*. *sonnei* are endemic and have been linked to most infections [10]. For both species, similar mortality rates [11] and frequencies of bacteremia per number of cases [5] have been reported. While *S*. *flexneri* is the most common cause of shigellosis, *S*. *sonnei* is replacing *S*. *flexneri* in locations where socio-economic conditions are improving and thus has become an important pathogen in developing countries [12,13]. In addition, *S*. *sonnei* bacteremia is likely to be underestimated as it is usually detected within the first 24 h of onset of disease when patients do not always seek medical attention [8].

*S*. *sonnei* comprises a single clonal group, characterized by low genetic variability and antigenic homogeneity [14,15]. All *S*. *sonnei* isolates have Phase I O somatic antigen, the immunodominant and protective antigen [16]. Phase I polysaccharide has an OAg repeating unit of two uncommon sugars not present in other *Shigella* serogroups, 2-acetamido-2-deoxy-L-altruronic acid (L-AltNAcA) and 2-acetamido-2-deoxy-L-fucose (FucNAc4N) [17]. For *Shigella* and genetically related *E*. *coli* species, LPS OAg biosynthesis is a Wzx/Wzy-dependent process, encoded by genes for synthesis of sugars of the repeating unit (called *wbg* cluster in *S*. *sonnei* [18] and *rfb* cluster in *S*. *flexneri* [19]), and for OAg unit transport (*wzx*) and polymerization (*wzy*) on the independently synthesized LPS core-lipid A moieties [9]. Unlike in other *Shigella*, the *S*. *sonnei wbg* OAg synthesis cluster is not located on the chromosome but on the *S*. *sonnei* large virulence plasmid (pSS) [20]. To invade and colonize the intestinal epithelium and to survive the strong inflammatory host response, *Shigella* requires expression of protein factors encoded by the virulence plasmid, such as the Type III Secretion System (T3SS) and its secreted effectors [1]. The LPS is also a key virulence determinant [21]. *S*. *flexneri* (2a and 5a) has LPS OAg with a bimodal chain length distribution which is regulated in a growth-dependent manner [22] and is important for bacterial mobility and serum resistance [23]. Moreover, phage-encoded glucosylation of the OAg is essential for optimized LPS and T3SS functions in *S*. *flexneri* 5a M90T [24]. Less is known about *S*. *sonnei* LPS: Phase I bacteria possess a single modal OAg with a predominant chain length of 20–25 units [18]. Expression of Phase I polysaccharide and virulence are strongly interconnected and loss of the pSS virulence plasmid *in vitro* results in the *S*. *sonnei* Phase II cell type, lacking both the OAg and the virulent phenotype [25,26].

Besides the OAg side chain of LPS, Gram-negative exopolysaccharides generally include other structures, e.g. capsules. These enhance the bacterial survival in the environment and their fitness within hosts, by avoiding elimination by innate immune killing [27]. *E*. *coli* capsules have historically been classified into four groups based on genetic and biochemical criteria [28]. Group 4 capsules (G4C) are comprised of a high molecular weight surface polysaccharide and are also known as ‘O antigen capsules’ due to their structural similarity to the OAg side chain of the LPS [28]. In a more recent classification distinguishing two major groups based on the primary mechanisms of biosynthesis, i.e. the ABC transporter-dependent and the Wzy-dependent capsular polysaccharides [29], group 4 capsules, together with group 1 capsules, belong to the Wzy-dependent group. OAg capsules have been found in intestinal pathogenic *E*. *coli*, such as enteropathogenic (EPEC) [30] and enterohemorrhagic *E*. *coli* (EHEC) [31], as well as in *Salmonella enterica* serovar Enteritidis [32], and have been shown to confer enhanced colonization [31] and environmental persistence [32]. G4C share the Wzy-dependent synthesis cluster for OAg units with the LPS, but require an additional *g4c* operon for secretion and assembly of the OAg polysaccharide from the periplasm into the capsular structure [28]. All seven genes of the *E*. *coli g4c* transcriptional unit (*ymcDCBA*, *yccZ*, *etp*, *etk*) were required for capsule production in EPEC serotype O127 [30]. Despite *Shigella* being classified as uncapsulated bacteria, genes homologous to the *E*. *coli g4c* operon are present in different strains [30], but expression of G4C as component of *Shigella* exopolysaccharide and its potential contribution to pathogenicity have not been described.

In this study, we show that *S*. *sonnei*, but not *S*. *flexneri* 2a, possesses a *g4c* operon-dependent OAg capsule. Deletion of the capsule in *S*. *sonnei* results in substantially increased invasiveness of HeLa cells *in vitro* and triggers increased inflammation in the intestine but decreases resistance to complement-mediated killing and spreading ability in the rabbit model. Thus, the *S*. *sonnei* G4C is an important factor in the regulation of pathogenesis and persistence.

## Results

### *S*. *sonnei* Δ*galU* lacking LPS-linked O antigen still has O antigen on the surface that is immunogenic and antigenic

**Fig 1:**
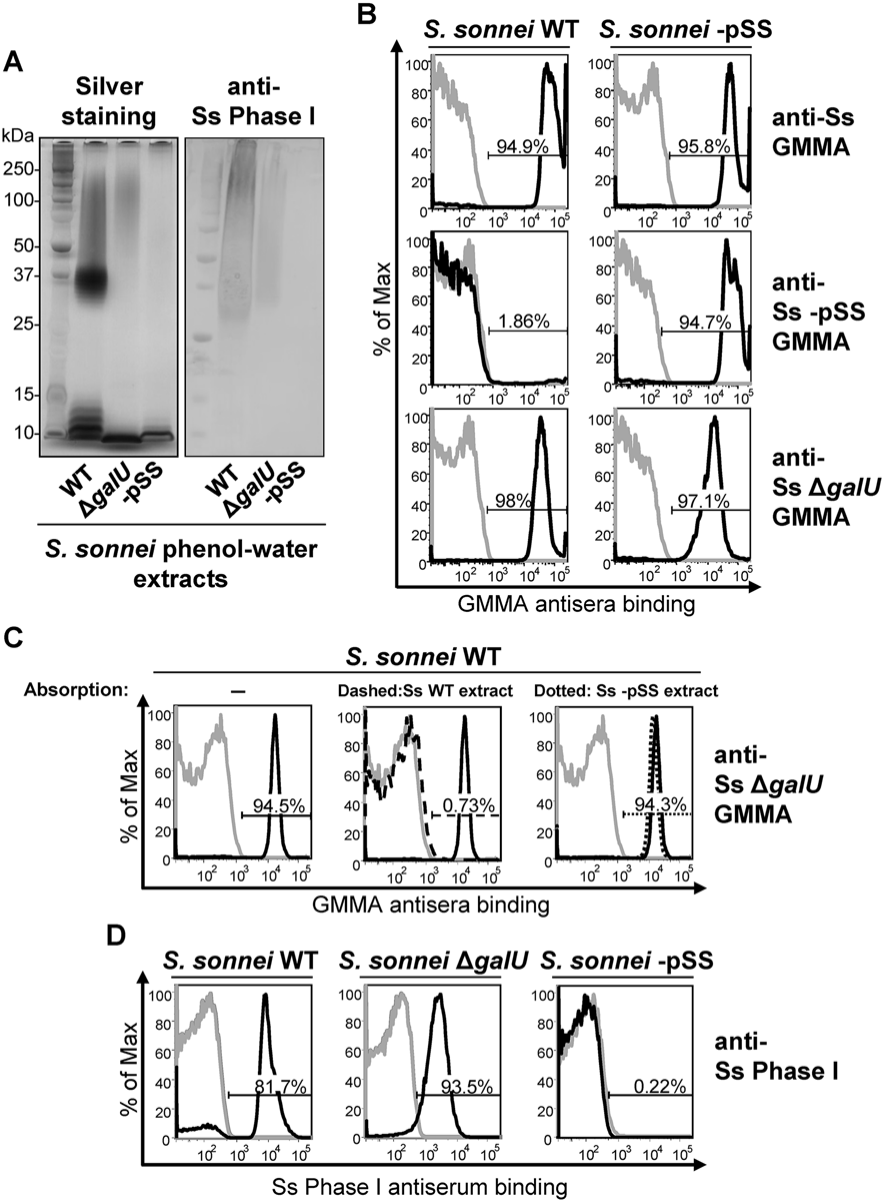
*S*. *sonnei* Δ*galU* LPS mutant possesses immunogenic and antigenic Phase I material on the surface. (**A**) Silver staining and immunoblot analysis of phenol-water extracts from *S*. *sonnei* WT, *S*. *sonnei* Δ*galU* and *S*. *sonnei* -pSS bacteria. Samples were run on 12% Bis-Tris SDS-PAGE, blotted, and membranes were incubated with *S*. *sonnei* Phase I monovalent antiserum (anti-Ss Phase I) at a dilution of 1:1000. (**B**) Flow cytometry analysis of surface staining of live *S*. *sonnei* WT and *S*. *sonnei* -pSS. Bacteria were stained with sera raised against GMMA from hyperblebbing *S*. *sonnei* with WT LPS (anti-Ss GMMA), *S*. *sonnei* -pSS with rough LPS (anti-Ss -pSS GMMA), or *S*. *sonnei* Δ*galU* with deep rough LPS (anti-Ss Δ*galU* GMMA). Gray profiles: staining with preimmune sera; black profiles: staining with GMMA antisera (1:1000). Representative results of three experiments are shown. (**C**) Competitive surface staining of *S*. *sonnei* WT live bacteria with sera raised against GMMA from hyperblebbing *S*. *sonnei* Δ*galU* strain (anti-Ss Δ*galU* GMMA). Gray profiles: staining with preimmune sera; black solid profiles: staining with anti-Ss Δ*galU* GMMA; black dashed profile: staining with anti-Ss Δ*galU* GMMA absorbed with *S*. *sonnei* WT (Ss WT) phenol-water extract; black dotted profile: staining with anti-Ss Δ*galU* GMMA absorbed with *S*. *sonnei* -pSS (Ss -pSS) phenol-water extract. Sera dilution was 1:10000. (**D**) Surface staining of formalin-fixed *S*. *sonnei* WT, *S*. *sonnei* Δ*galU* and *S*. *sonnei* -pSS with *S*. *sonnei* Phase I monovalent antiserum (anti-Ss Phase I). Gray profiles: staining with *S*. *flexneri* type II monovalent antiserum; black profiles: staining with anti-Ss Phase I. Sera dilution was 1:5000.

By SDS-PAGE (Fig. 1A), the phenol-water extract of *S*. *sonnei* WT had a typical LPS ladder with a predominant chain length of 20 to 25 OAg repeating units [18] and additional lower mobility material above the position of 25 units. *S*. *sonnei* strain lacking the pSS OAg-encoding virulence plasmid (*S*. *sonnei* -pSS) had only the low molecular weight band corresponding to the LPS core-lipid A moieties. *S*. *sonnei* Δ*galU* is a deep rough LPS mutant with a defect in the pathway that transfers the OAg onto the LPS core region [21]. Its extract had slowly migrating material in addition to the low molecular weight band of the LPS inner core-lipid A molecules, but no LPS ladder. A Western blot probed with a monovalent anti-*S*. *sonnei* Phase I typing serum displayed a band with similar low mobility in phenol-water extracts of *S*. *sonnei* WT and *S*. *sonnei* Δ*galU* but not in *S*. *sonnei* -pSS extract (Fig. 1A). To immunize mice, we generated outer membrane particles, called Generalized Modules for Membrane Antigens (GMMA), from *S*. *sonnei* WT, *S*. *sonnei* Δ*galU* and *S*. *sonnei-*pSS strains genetically modified to induce high level shedding of these particles (hyperblebbing) by deletion of the *tolR* gene [33]. GMMA are highly immunogenic and present surface antigens in their natural context. By flow cytometry (Fig. 1B), sera raised against *S*. *sonnei* GMMA with unmodified LPS reacted with *S*. *sonnei* WT and *S*. *sonnei* -pSS bacteria. Sera raised against *S*. *sonnei* -pSS GMMA reacted with *S*. *sonnei* -pSS but not with *S*. *sonnei* WT, indicating that the OAg on the target *S*. *sonnei* WT bacteria shields the surface antigens from the antibodies raised against OAg-negative GMMA. Sera raised against *S*. *sonnei* Δ*galU* GMMA reacted like sera raised against *S*. *sonnei* GMMA with unmodified LPS, staining *S*. *sonnei* WT and *S*. *sonnei* -pSS bacteria, suggesting that they had anti-OAg activity despite lacking LPS-linked OAg. This immunoreactivity was not an artifact of GMMA immunization, as sera raised against whole formalin-fixed *S*. *sonnei* WT, *S*. *sonnei* with a knockout of the *wbg* OAg biosynthesis cluster in the pSS virulence plasmid (*S*. *sonnei* ΔOAg), and *S*. *sonnei* Δ*galU* bacteria gave similar results (S1 Fig). Unlike *S*. *sonnei* Δ*galU*, sera raised against whole inactivated *S*. *flexneri* 2a Δ*galU* did not react with its homologous *S*. *flexneri* 2a WT strain, indicating the lack of OAg-specific immunogenicity in this background (S1 Fig).

We performed a competitive staining experiment (Fig. 1C) to test if OAg-specific antibodies in *S*. *sonnei* Δ*galU* GMMA antisera were responsible for the binding to *S*. *sonnei* WT. Pre-absorption of anti-*S*. *sonnei* Δ*galU* GMMA sera with phenol-water extract from *S*. *sonnei* WT but not from *S*. *sonnei* -pSS bacteria abolished reactivity (Fig. 1C). In addition, flow cytometry of formalin-fixed *S*. *sonnei* WT, Δ*galU* and -pSS bacteria stained with the *S*. *sonnei* Phase I typing antiserum gave strong signals with the WT and the Δ*galU* strains, while no binding was revealed on the control *S*. *sonnei* -pSS strain (Fig. 1D). These results confirm that *S*. *sonnei* Δ*galU* expresses an OAg material on the surface. However, the Δ*galU* OAg may be loosely attached to the outer membrane since the anti-Phase I surface staining of live *S*. *sonnei* Δ*galU* target bacteria was variable and much less pronounced (S1 Table).

### *S*. *sonnei g4c* operon encodes the high molecular weight O antigen capsule

Genomic analysis showed a gene cluster in *S*. *sonnei* 53G and 046 with ≥ 99% identity to the *E*. *coli g4c* operon in the coding regions and the same genetic orientation (*ymcDCBA*, *yccZ*, *etp*, *etk*) (S2 Fig). Homologous genes are also present in *S*. *flexneri* 2a 2457T and 301 genomes, but a deletion of 14 bases (TGTCGCTTACTCGC) in the *etk* locus (position 135 to 148) causes a frame-shift mutation and thus inactivation of the operon (S2 Fig).

**Fig 2:**
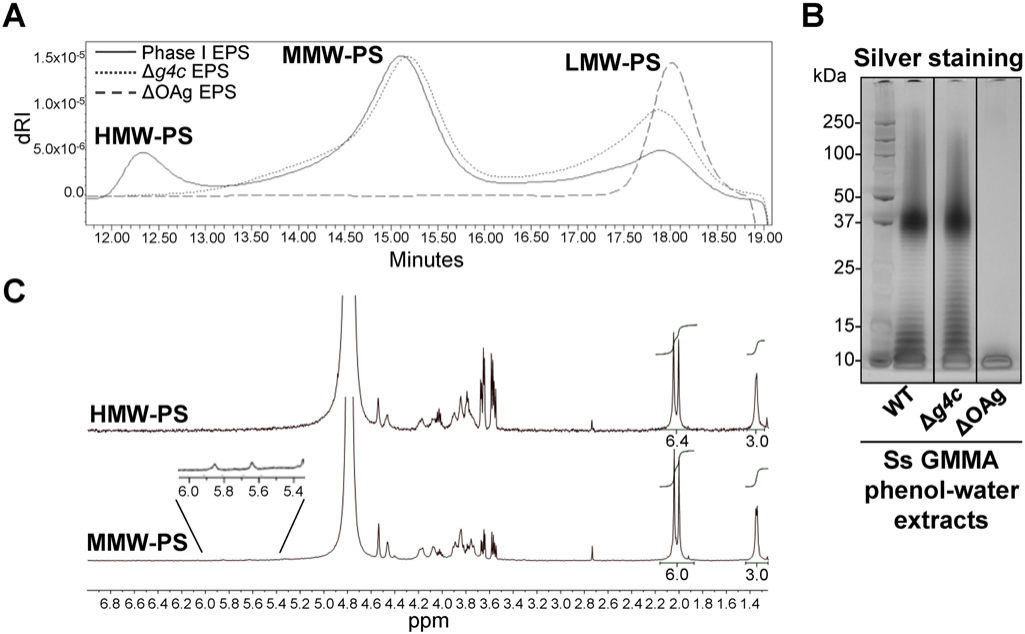
*S*. *sonnei g4c* cluster encodes for a high molecular weight O antigen polysaccharide. (**A**) HPLC-SEC (dRI) analysis showing molecular weight distribution (high, medium, and low molecular weight polysaccharides, respectively HMW, MMW, LMW-PS) of acid-cleaved exopolysaccharide (EPS) purified from GMMA of hyperblebbing *S*. *sonnei* (Phase I EPS, solid line), *S*. *sonnei* Δ*g4c* (Δ*g4c* EPS, dotted line) and *S*. *sonnei* ΔOAg (ΔOAg EPS, dashed line). Polysaccharide samples were run on TosoHaas TSK gel G3000 PWXL-CP column (distribution coefficients (Kd): Kd_HMW-PS_ = 0.09, Kd_MMW-PS_ = 0.31). Apparent average molecular weight of HMW-PS (197.49 kDa) and of MMW-PS (22.04 kDa) was estimated by running Phase I EPS with a dextran standard curve. (**B**) 12% Bis-Tris SDS-PAGE and silver staining of *S*. *sonnei* (Ss) phenol-water extracts from GMMA of hyperblebbing *S*. *sonnei* (WT EPS), *S*. *sonnei* Δ*g4c* (EPS lacking HMW-PS), and *S*. *sonnei* ΔOAg (only LMW-PS). (**C**) ^1^H-NMR spectra of *S*. *sonnei* Phase I EPS populations. Integral of the signals at 2.00 and 2.04 ppm belonging to the N-acetyl groups of the FucNAc4N and the L-AltNAcA OAg residues and of the signal at 1.34–1.36 ppm of the FucNAc4N methyl group are reported. Region of anomeric signals of terminal and internal α-galactose residues from LPS outer core in MMW-PS (5.82 ppm and 5.62 ppm, respectively) is shown enlarged.

A *S*. *sonnei g4c* mutant strain (*S*. *sonnei* Δ*g4c*) was generated to assess the functionality of the cluster. Furthermore, we inserted a selectable marker into the OAg-encoding pSS (replacement of *virG* by a resistance gene) to avoid loss of the plasmid during *in vitro* culture, and thereby obtained *S*. *sonnei* strains with stable OAg expression. These modifications were also introduced into hyperblebbing strains with the aim to use GMMA as source of exopolysaccharides for biochemical analyses. GMMA mainly contain outer membrane components and have negligible amounts of nucleic acids and cytoplasmic impurities [33]. We found acid-cleaved phenol-water exopolysaccharide extracts from GMMA to be suitable for polysaccharide analysis without need for further purification. Exopolysaccharide molecular weight distribution was examined by HPLC-SEC (Fig. 2A). The Phase I exopolysaccharide (Fig. 2A, solid line) from *S*. *sonnei* with unmodified LPS possessed a trimodal distribution of low, medium and high molecular weight (LMW, MMW, HMW, respectively) polysaccharides. A single LMW polysaccharide population was obtained from *S*. *sonnei* ΔOAg exopolysaccharide (Fig. 2A, dashed line). Exopolysaccharide from *S*. *sonnei* Δ*g4c* had similar MMW and LMW populations as the *S*. *sonnei* Phase I exopolysaccharide, but no HMW polysaccharide (Fig. 2A, dotted line). Silver stained SDS-PAGE of the corresponding phenol-water GMMA extracts (Fig. 2B) correlated with the HPLC-SEC analysis but did not differentiate between the patterns from *S*. *sonnei* with trimodal WT exopolysaccharide and from *S*. *sonnei* Δ*g4c* deficient for the HMW polysaccharide. In *S*. *sonnei* Δ*galU* exopolysaccharide, only the HMW polysaccharide and a very LMW polysaccharide were present (S3 Fig). The Phase I exopolysaccharide was fractionated by HPLC-SEC and eluted fractions were analyzed by ^1^H-NMR (Fig. 2C). Spectra of the HMW and the MMW polysaccharides had peaks from the FucNAc4N and the L-AltNAcA residues of the *S*. *sonnei* OAg [34]. Integration of these signals confirmed the 1:1 ratio between FucNAc4N and L-AltNAcA expected for the *S*. *sonnei* OAg units. Anomeric signals of terminal and internal α-galactose residues from the outer core of *S*. *sonnei* LPS (5.82 ppm and 5.62 ppm, respectively) [34] were detected in the MMW polysaccharide. From the ratio between signals of the N-acetyl groups of the OAg residues (2.00–2.04 ppm) and signals of the outer core α-galactose residues (5.82 and 5.62 ppm), we calculated that the MMW polysaccharide has about 23–30 OAg units, in line with the SDS gels and literature values for the main LPS population [18]. The size of the HMW polysaccharide was estimated by HPLC-SEC in comparison with a dextran standard curve at 206–269 OAg units. To assess the presence of LPS core in the different polysaccharide populations, we analyzed the content of core residue 3-deoxy-D-manno-octulosonic acid (KDO) after acid hydrolysis at the reducing end [35]. KDO was detected in the LMW and MMW polysaccharide, but not in the capsular HMW polysaccharide (S4 Fig). Thus, the *g4c* operon is functional in *S*. *sonnei* and encodes for the formation of an LPS-unlinked high molecular weight OAg polysaccharide, i.e. a group 4 capsule.

**Fig 3:**
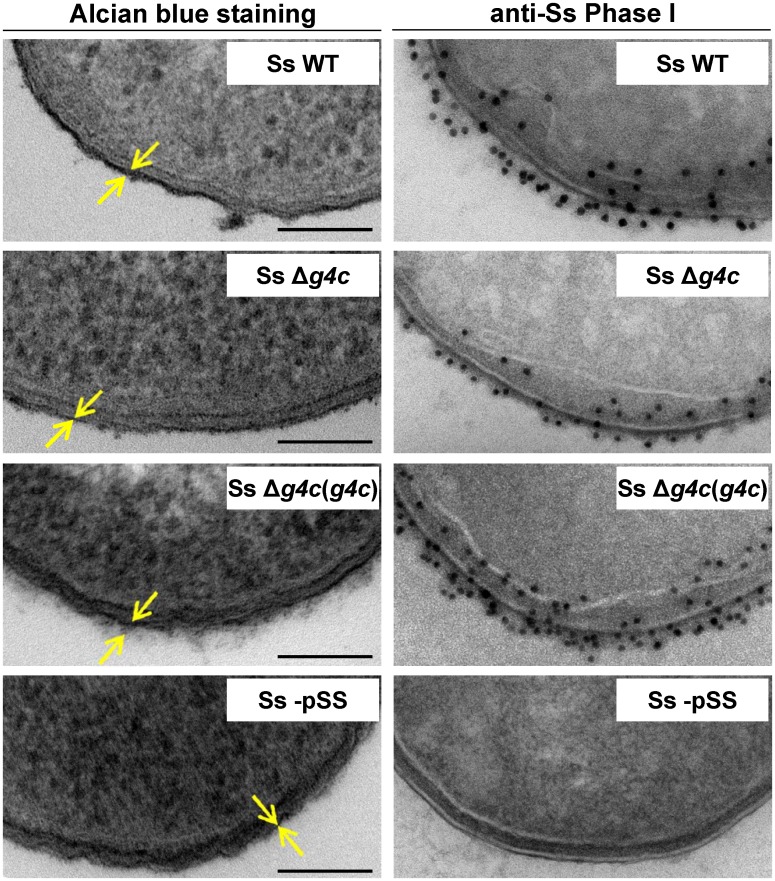
Group 4 capsule forms a dense outer layer on the surface of *S*. *sonnei*. Surface analysis of *S*. *sonnei* WT (Ss WT), *S*. *sonnei* Δ*g4c* (Ss Δ*g4c*), *S*. *sonnei* Δ*g4c*(*g4c*) (Ss Δ*g4c*(*g4c*)) and *S*. *sonnei* -pSS (Ss -pSS) bacteria by electron microscopy. Left column: electron-dense material at the bacterial surface corresponding to exopolysaccharides is revealed by alcian blue staining. Scale bar: 100 nm. Arrows indicate the LPS/capsule layer. Right column: immunogold labelling of *S*. *sonnei* bacteria with anti-*S*. *sonnei* Phase I monovalent antiserum (anti-Ss Phase I).

Transmission electron microscopy of alcian blue stained *S*. *sonnei* WT displayed a dark layer of electron-dense material corresponding to the exopolysaccharides (Fig. 3). Immunogold staining using anti-Phase I serum localized the Phase I antigens on the external surface of the outer membrane, protruding about 10–20 nm, and within the periplasmic space. In the absence of the G4C, *S*. *sonnei* Δ*g4c* bacteria possessed an OAg layer of about half of the thickness of the *S*. *sonnei* WT layer. G4C expression in *S*. *sonnei* Δ*g4c* was restored by complementing the knockout *in trans* through the insertion of a functional operon with its own promoter on a low copy number vector. In these *S*. *sonnei* Δ*g4c*(*g4c*) bacteria, the thickness of the Phase I layer was augmented, extending from the outer membrane about 20–30 nm. The control *S*. *sonnei* -pSS OAg-deficient strain was negative for both the alcian blue and the immune labelling, confirming staining specificity.

### *S*. *sonnei* entry into epithelial cells is affected by the group 4 capsule

**Fig 4:**
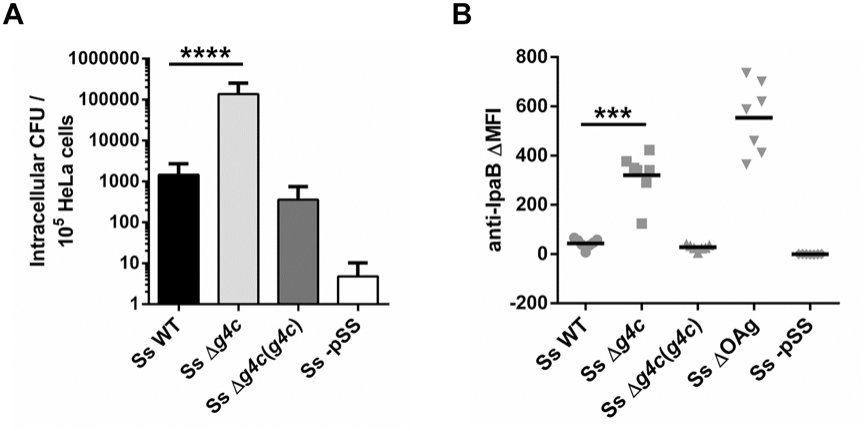
Group 4 capsule impacts *S*. *sonnei* HeLa cell invasion and IpaB exposure on the surface. (**A**) HeLa epithelial cells were infected with *S*. *sonnei* WT (Ss WT), *S*. *sonnei* Δ*g4c* (Ss Δ*g4c*), *S*. *sonnei* Δ*g4c*(*g4c*) (Ss Δ*g4c*(*g4c*)) or *S*. *sonnei* -pSS (Ss -pSS) at an MOI of 10. Columns show the average number and standard deviation (error bars) of intracellular bacteria (CFU) collected from 10^5^ cells in three independent experiments in triplicate (****p<0.0001, Mann-Whitney test). (**B**) Flow cytometry analysis of IpaB exposure on the surface of live *S*. *sonnei* WT (Ss WT), *S*. *sonnei* Δ*g4c* (Ss Δ*g4c*), *S*. *sonnei* Δ*g4c*(*g4c*) (Ss Δ*g4c*(*g4c*)), *S*. *sonnei* ΔOAg (Ss ΔOAg) and *S*. *sonnei* -pSS (Ss -pSS). Results are expressed as differential Mean Fluorescence Intensity (ΔMFI): the difference between the MFI of the anti-IpaB immune straining and the MFI of a control staining, using only the secondary antibody. Three independent experiments were performed, twice in triplicate and once as single assay. Individual results are shown as scatter plot, the mean ΔMFI is shown as line (***p = 0.0006, Mann-Whitney test).

The invasiveness of *S*. *sonnei* WT and *S*. *sonnei* Δ*g4c* was investigated *in vitro* by evaluating the number of intracellular bacteria after 1 h infection in HeLa cells (Fig. 4A). Uncapsulated *S*. *sonnei* Δ*g4c* had about 100-fold more colony forming units (CFU) compared to the WT strain. Complementation of the *g4c* knockout in *S*. *sonnei* Δ*g4c*(*g4c*) reduced invasiveness of *S*. *sonnei* Δ*g4c* by about 400-fold, resulting in 4-fold less intracellular bacteria than with *S*. *sonnei* WT. *S*. *sonnei* -pSS lacking the virulence plasmid did not invade HeLa cells, as expected [25]. These results show that the expression level of the capsule polysaccharide affects *S*. *sonnei* cell invasion *in vitro*. Thus, we investigated if the G4C masks critical elements of the invasion complex. Using flow cytometry (Fig. 4B) we examined the ability of a monoclonal antibody to bind to the invasion plasmid antigen B (IpaB) located at the tip of T3SS [36]. IpaB-dependent fluorescence on the surface of *S*. *sonnei* WT and *S*. *sonnei* Δ*g4c*(*g4c*) was only detected at a level comparable to the negative control. The anti-IpaB signal was stronger on the capsule-deficient *S*. *sonnei* Δ*g4c* strain. By Western blot, similar amounts of IpaB protein were detected in *S*. *sonnei* WT and *S*. *sonnei* Δ*g4c* (S5 Fig). Thus, the T3SS tip is more accessible in the absence of the capsule. IpaB was not detected by Western blot in *S*. *sonnei* Δ*g4c*(*g4c*) suggesting an interference of the excess of capsule material either with IpaB detection or with IpaB expression. The anti-IpaB staining on *S*. *sonnei* ΔOAg, lacking both the LPS OAg side chain and the G4C but still possessing the virulence plasmid, was further increased over the staining on uncapsulated *S*. *sonnei* Δ*g4c*. *S*. *sonnei* -pSS was negative, as expected.

### Uncapsulated *S*. *sonnei* Δ*g4c* triggers increased inflammation in the rabbit intestine but has decreased spreading ability and complement resistance

**Fig 5:**
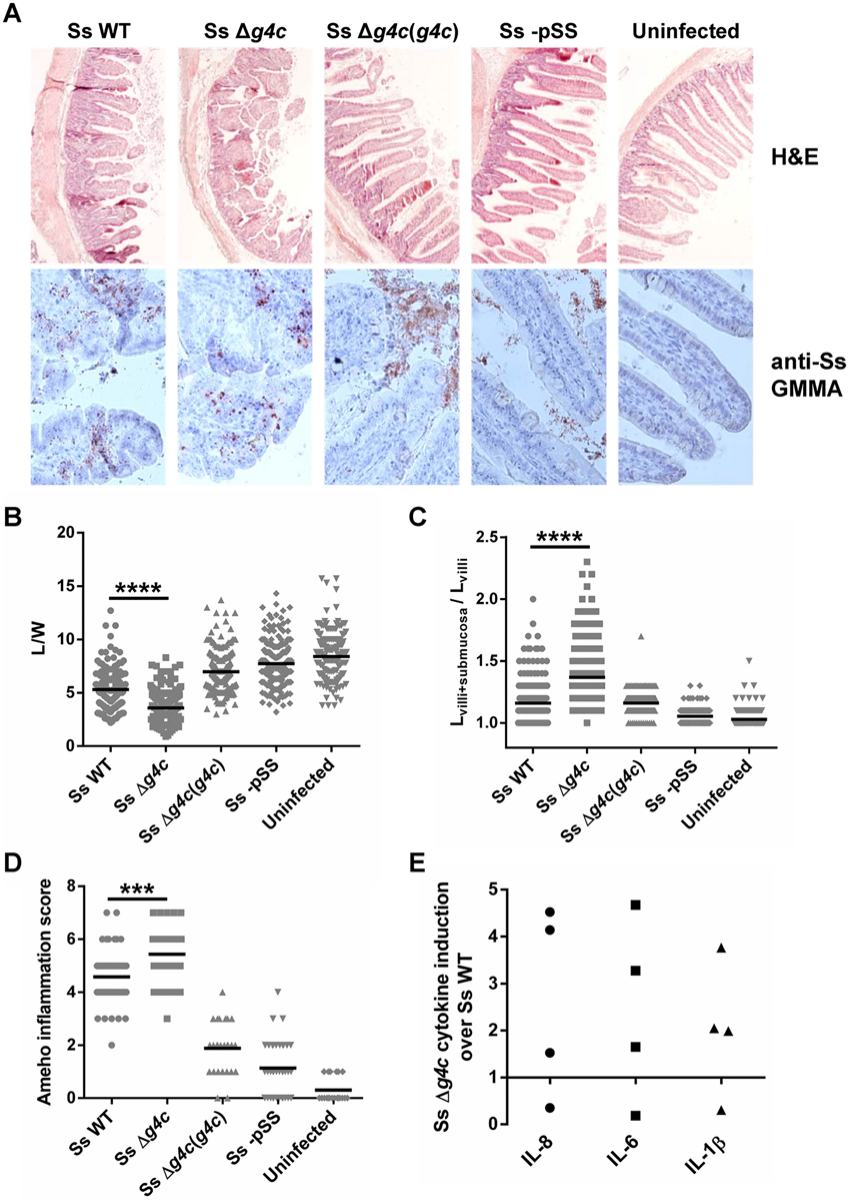
Group 4 capsule affects the pattern of *S*. *sonnei* pathology in the rabbit intestine. (**A**) Representative results of histopathological analysis of *S*. *sonnei* WT (Ss WT), *S*. *sonnei* Δ*g4c* (Ss Δ*g4c*), *S*. *sonnei* Δ*g4c*(*g4c*) (Ss Δ*g4c*(*g4c*)) and *S*. *sonnei* -pSS (Ss -pSS) infected loops after 8 h of single strain challenge. Control sample is the uninfected tissue. Upper row: Hematoxylin and Eosin (H&E) staining of 7 μm slices of rabbit ileal loops. Lower row: immunostaining of 7 μm slices of rabbit loops with a polyclonal *S*. *sonnei* GMMA antisera (anti-Ss GMMA) to demonstrate bacteria localization (red dots) and counterstaining with Hematoxylin. (**B**) Histological alteration observed in the rabbit model following 8 h infection by *S*. *sonnei* strains. Severity of villi atrophy is measured according to the ratio between the length and width (L/W) of 120 villi counted on each among at least 4 different loops in 2 different animals (****p<0.0001, Mann-Whitney test). (**C**) Severity of submucosal edema is measured according to the ratio between the length of the entire mucosal and submucosal layer and the length of the villi (L_villi+submucosa_/L_villi_). 120 measurements were performed on each among at least 4 different loops in 2 different animals (****p<0.0001, Mann-Whitney test). (**D**) Evaluation of the *Shigella*-induced pathology for each strain according to the Ameho histopathological grading scale was performed on at least 4 image fields of 4 different loops in 2 different animals (***p = 0.0001, Mann-Whitney test). (**E**) Induction of IL-8, IL-6 and IL-1β in rabbit loops by *S*. *sonnei* Δ*g4c* (Ss Δ*g4c*) relative to *S*. *sonnei* WT (Ss WT). Expression levels were measured by RT-qPCR in the set up experiment using an infectious dose of 5x10^9^/loop (the other experiments used 3x10^9^ bacteria/loop). Two loops per strain were inoculated in 2 different animals. The scatter plot shows induction of gene expression in *S*. *sonnei* Δ*g4c* loops relative to the adjacent *S*. *sonnei* WT loop.

The impact of the capsule on *S*. *sonnei* virulence was tested in the rabbit ligated ileal loop model. Separate intestinal loops were infected with equal numbers (3x10^9^) of *S*. *sonnei* WT, capsule-deficient *S*. *sonnei* Δ*g4c*, complemented *S*. *sonnei* Δ*g4c*(*g4c*), or *S*. *sonnei* -pSS per loop (3 replicate loops per animal for each strain). Eight hours after infection, local sites were examined for *Shigella*-dependent pathology, while peripheral sites were examined for the relative presence of the different *S*. *sonnei* strains. In the intestine, fluids and blood were present in the *S*. *sonnei* WT-infected loops. *S*. *sonnei* Δ*g4c* caused more fluid and blood accumulation, with loops having pale outer surface and intense inflammation. The pathology observed with the complemented *S*. *sonnei* Δ*g4c*(*g4c*) strain was less severe than that with the WT, whereas the non-invasive *S*. *sonnei* -pSS barely caused fluid production and tissue alteration. Histopathology supported the qualitative data (Fig. 5A). Hematoxylin and Eosin (H&E) stained slices showed alterations of villi from infected tissues and bacteria were detected by immunostaining with a polyclonal anti-*S*. *sonnei* GMMA serum. After infection by *S*. *sonnei* WT, villi were shortened and enlarged, with an average length to width ratio (L/W) of 5.3 (Fig. 5B), with several indentations. Numerous regions of tissue disruption were observed and infiltration of inflammatory cells was detected within the lamina propria and in the edematous submucosal tissues (Fig. 5C). These observations resulted in an inflammation score of 4.6, according to the Ameho criteria with grading scores from 0–6 [37] (Fig. 5D). Infection by *S*. *sonnei* Δ*g4c* led to dramatic alterations of mucosal tissues with extensive zones of rupture and destruction of the intestinal epithelium, including epithelial detachment and loss of villi with tissue necrosis. Remaining villi had a lower L/W ratio (3.6) than villi in *S*. *sonnei* WT-infected loops (Fig. 5B). Submucosal tissues were strongly edematous (Fig. 5C), with a large area infiltrated by inflammatory cells between the residual mucosa and the muscular layer. These observations were graded as a very high 5.4 Ameho score (Fig. 5D). In accordance with the profound epithelial changes, following infection with both *S*. *sonnei* WT and *S*. *sonnei* Δ*g4c*, most bacteria were associated with the lamina propria and the epithelium of the villi, particularly in areas of abscesses, rupture/destruction of the epithelial lining and of villi indentation (Fig. 5A). In contrast, slices from *S*. *sonnei* Δ*g4c*(*g4c*)-infected tissues showed a lower level of pathology (Ameho score of 1.9), with villi with a length to width ratio (L/W of 7.0) similar to those following infection with *S*. *sonnei* -pSS or uninfected (L/W of 7.7 and 8.4, respectively), limited edema with few cells infiltrating the lamina propria, and few tissue lesions. The reduced severity of tissue damage in H&E-stained slices correlated with most of the *S*. *sonnei* Δ*g4c*(*g4c*) bacteria being present in the lumen instead of in the epithelium. Villi of loops infected with the avirulent *S*. *sonnei* -pSS strain were long and narrow, similar to the normal rabbit epithelial architecture of uninfected tissue, with no evidence of mucosal alteration, a low inflammation score of 1.1, and no invasive bacteria detected within the epithelium.

Pro-inflammatory cytokines were measured to further analyze the inflammatory response of the tissue in the infected loops. In an initial experiment to establish the infectious dose of *S*. *sonnei* in the rabbit ligated loop model, 5x10^9^ bacteria per loop were used. At this dose, the *S*. *sonnei* Δ*g4c* capsule-deficient strain induced, on average, higher levels of Interleukin 8 (IL-8), IL-6, and IL-1β compared to *S*. *sonnei* WT (Fig. 5E) but the number of replicate loops tested (2 each in 2 rabbits) was too small to reach statistical significance. The high induction of pro-inflammatory cytokines was accompanied by severe tissue destruction. Thus, for the main experiment a slightly lower infectious dose (3x10^9^/loop) was chosen. At this dose, in general lower inflammation was observed. While the histology assessment showed a difference in the inflammatory potential of *S*. *sonnei* WT and Δ*g4c* as described above, no difference was detected in the induction of pro-inflammatory cytokines, possibly since, at the lower dose, lesions are more dispersed and the analysis is performed on whole loops so that the results from the infected tissue are hidden by the results from the normal tissue.

**Fig 6:**
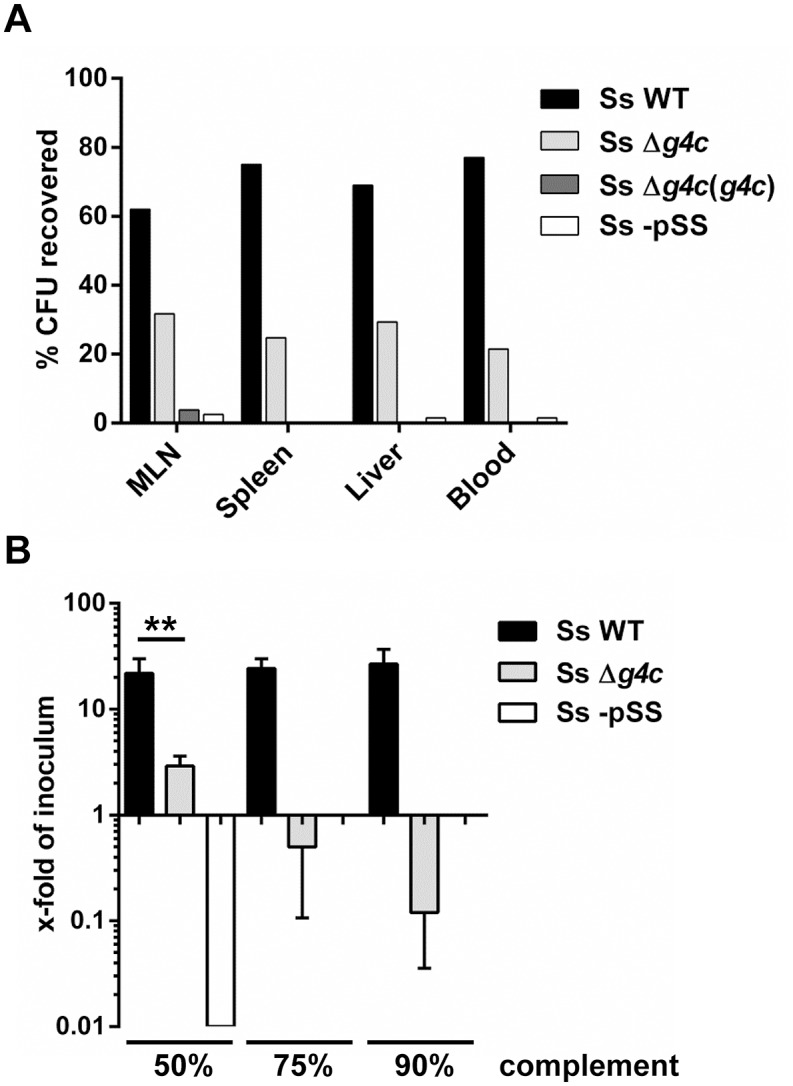
Group 4 capsule affects *S*. *sonnei* peripheral spreading and sensitivity to direct complement lysis. (**A**) Systemic load of *S*. *sonnei* WT (Ss WT), *S*. *sonnei* Δ*g4c* (Ss Δ*g4c*), *S*. *sonnei* Δ*g4c*(*g4c*) (Ss Δ*g4c*(*g4c*)) and *S*. *sonnei* -pSS (Ss -pSS) bacteria following 8 h of single strain infection in separate loops (3 loops per strain) in the same animal. The different strains were enumerated by plating on solid media using the distinct antibiotic resistance profiles of the strains for their differentiation. The prevalence of individual strains in mesenteric lymph nodes (MLN), spleen, liver, or blood is reported as percent of CFU recovered from the specific organ. Results present the average counts of 2 animals. (**B**) Sensitivity of *S*. *sonnei* WT (Ss WT), *S*. *sonnei* Δ*g4c* (Ss Δ*g4c*) and *S*. *sonnei* -pSS (Ss -pSS) strains to increasing concentrations of baby rabbit complement (50, 75, and 90%). Assays with 3 h incubation were performed in triplicate in three independent experiments and the results are expressed as x-fold increase/decrease compared to the number of the bacteria in the inoculum. No colonies were retrieved after incubation of *S*. *sonnei* -pSS with 75% and 90% of complement. (**p = 0.0022, Mann-Whitney test).

In the same model used for assessing the *Shigella*-induced histopathology, we then investigated how the G4C contributes to *S*. *sonnei* dissemination by evaluating the bacterial load in mesenteric lymph nodes, spleen, liver, or blood. Eight hours after infection of separate intestinal loops in the same animal with *S*. *sonnei* WT, *S*. *sonnei* Δ*g4c*, *S*. *sonnei* Δ*g4c*(*g4c*), or *S*. *sonnei-*pSS the number of bacteria from these strains in the systemic organs was determined (Fig. 6A). In each organ *S*. *sonnei* WT bacteria predominated (on average 70% of the bacteria recovered in the specific organ). *S*. *sonnei* Δ*g4c* bacteria accounted for 26% of bacteria per organ, and only a negligible number of *S*. *sonnei* -pSS and *S*. *sonnei* Δ*g4c*(*g4c*) bacteria was found peripherally.

Sensitivity to complement was assessed (Fig. 6B) as a measure for the ability of *S*. *sonnei* WT and uncapsulated *S*. *sonnei* Δ*g4c* to survive in the systemic environment. *S*. *sonnei* WT was highly resistant to complement: even in 90% baby rabbit complement, the number of cells increased to 22-fold of the inoculum size during 3 h incubation (approximately 4.5 generations). In contrast, in 50% baby rabbit complement the number of cells of the capsule-deficient strain increased to only 3-fold of inoculum size and 90% baby rabbit complement resulted in killing of the mutant to a viable cell count of 10% of the inoculum. *S*. *sonnei* -pSS and *S*. *flexneri* 2a served as controls. As previously reported [38,39], *S*. *sonnei* -pSS was not able to survive in 50% complement (Fig. 6B), and *S*. *flexneri* 2a was more sensitive to complement than *S*. *sonnei* WT (S6 Fig). Thus, while the OAg is essential for resistance to serum lysis [38] the capsule strongly augments *S*. *sonnei* resistance to direct complement-mediated killing.

Collectively, these data show that the lack of capsule increases *S*. *sonnei* local pathogenicity but reduces peripheral dissemination, likely due to reduced complement resistance. Conversely, overexpression of capsule (*S*. *sonnei* Δ*g4c*(*g4c*)) decreased dissemination, presumably because these bacteria failed to invade epithelial cells effectively.

## Discussion

The presence of a group 4 capsule in *S*. *sonnei* was uncovered by the immunological characterization of the *S*. *sonnei galU* knockout deep rough mutant that does not possess an acceptor for the OAg polysaccharide on the LPS molecule. We demonstrated that this mutant still has the ability to assemble OAg units on the surface through an alternative pathway to form an OAg capsule, in accordance with the presence of an intact *wbg* OAg (Wzy-dependent [29]) synthesis cluster on the *S*. *sonnei* virulence plasmid, essential for both LPS OAg and OAg capsule. A low mobility material, not linked to the LPS, was extracted from *S*. *sonnei* WT and Δ*galU* bacteria and was visualized in immunoblot analyses with a Phase I-specific antiserum. This observation was similar to the identification of a slowly migrating Phase I-reactive material in *S*. *sonnei* and in a Phase I-transformed *Salmonella* Typhi live vector vaccine candidate [18]. Although an OAg capsule-like material in *S*. *sonnei* has been suggested [40], no further studies have clarified this hypothesis. We found that SDS-PAGE analyses could not differentiate between LPS alone and LPS plus capsule, with both running with a smearing component. Thus, in the present study the identity of the *S*. *sonnei* G4C was confirmed by genetic and structural analyses. Genes homologous to those identified in *E*. *coli* for the G4C were found in different *Shigella* genomes [30]. In agreement with this finding, *S*. *sonnei* strains 53G and 046 were confirmed to have an intact *g4c* operon and its functionality in *S*. *sonnei* 53G was demonstrated. In contrast, our genomic analysis revealed that *S*. *flexneri* 2a strains 2457T and 301 carry an inactivated operon due to a deletion in the *etk* locus. Consequently, in *S*. *flexneri* 2a the Δ*galU* mutation resulted in OAg deficiency as previously reported [21], as the strain lacks the capsule assembly system that would provide an alternative pathway for OAg surface expression to LPS.

We studied the biochemical characteristics of the *S*. *sonnei* G4C extracted from GMMA since these outer membrane preparations contain very low amounts of DNA and cytoplasmic components. We used G4C positive and negative isogenic mutants from hyperblebbing *S*. *sonnei* strains with stabilized virulence plasmid-driven expression of the OAg to obtain highly purified exopolysaccharide and demonstrated that the *g4c* cluster encodes for the formation of a high molecular weight polysaccharide with about 10 times higher molecular weight than the main medium molecular weight LPS population. Phase I antigen comprising both, LPS OAg and OAg capsule, is likely to be a common feature in the *S*. *sonnei* serogroup. For instance, a similar exopolysaccharide profile to *S*. *sonnei* 53G was found in a different *S*. *sonnei* isolate (25931, S2 Table). The LPS OAg and the capsular OAg share the same biosynthesis cluster. Accordingly, mutants in the *wbg* OAg operon (*S*. *sonnei* -pSS and *S*. *sonnei* ΔOAg) were both rough and uncapsulated. LPS core residues were found in the LPS-derived medium and low molecular weight polysaccharides, but not in the capsular polysaccharide, suggesting again that this population is not a high molecular weight LPS species, but is linked to the bacteria independently of the LPS. The *S*. *sonnei* Phase I polysaccharide has an uncommon zwitterionic structure [41] of sugars carrying a carboxylic group (C-4 of the FucNAc4N) and an amino group (C-6 of the L-AltNAcA) [9]. Given the presence of amino groups in the OAg units, fixation with formaldehyde is likely to cross-link the capsule and to bind it to LPS OAg and to surface proteins, making its detachment from the bacteria more difficult, as it was shown in EM and flow cytometry analyses. However, we found G4C bound to unfixed bacteria and on GMMA indicating that *S*. *sonnei* G4C is not completely released by bacteria. Therefore, the presence of a linker molecule could be envisaged. Structural analyses of G4C from *Salmonella* Enteritidis have shown that purified capsule has fatty acids at levels consistent with a lipid anchor [42]. Further studies are needed to test if the *S*. *sonnei* capsule is linked to the surface through such an anchor.

The current model of *Shigella* pathogenesis is mainly derived from studies of *S*. *flexneri* [1]. *S*. *flexneri* has a bimodal LPS OAg distribution including the so-called very long OAg that contributes to virulence in *S*. *flexneri* 2a [23] and is absent in *S*. *sonnei* [18]. Instead, as *S*. *sonnei* but not *S*. *flexneri* 2a was found to be encapsulated, we investigated the role of G4C as a *S*. *sonnei* specific virulence factor. We demonstrated that *S*. *sonnei* G4C, together with the LPS OAg, constitutes a dense outer layer that impacts on *S*. *sonnei* surface accessibility and invasive potential. For example, the exopolysaccharide layer masked antibodies raised against OAg-lacking outer membrane GMMA particles from recognizing their target epitopes *in vitro*. Bacterial capsules have been characterized to modulate the functionality of other virulence factors, such as adhesins in *E*. *coli* [43], fimbriae in *Klebsiella* [44] and pili in *Neisseria* [45]. G4C, in particular, have been shown to play critical roles in interactions between bacteria and their immediate environments [32] and hosts [31]. In pathogenic *E*. *coli*, G4C appeared to mask surface structures, inhibit the attachment of bacteria to tissue-cultured epithelial cells, diminish their capacity to induce the formation of actin pedestals, and attenuate T3SS-mediated protein translocation into host cells [31]. Similarly, in *S*. *sonnei* the presence of the capsule polysaccharide accounted for changes in cell invasion ability *in vitro*. Uncapsulated *S*. *sonnei* Δ*g4c* were significantly more invasive than *S*. *sonnei* WT, while capsule overexpressing *S*. *sonnei* Δ*g4c*(*g4c*) strain displayed reduced cell entry. Thus, the *in vitro* studies indicated a role of the G4C in negatively affecting the invasive abilities of *S*. *sonnei*. The virulence of *Shigella* is mainly mediated by the activity of an array of plasmid-encoded virulence factors among which the T3SS is one of the most important [1]. We evaluated the possibility of capsule-mediated shielding of T3SS in *S*. *sonnei*. We observed that the accessibility of the IpaB protein at the tip of the T3SS was increased *in vitro* in *S*. *sonnei* Δ*g4c* compared to the WT strain. As similar amounts of IpaB were detected by Western blot this showed that the G4C at least partially covers the tip of the T3SS. The importance of a balance between surface LPS OAg length and T3SS exposure in *Shigella* has been demonstrated for *S*. *flexneri* 5a M90T, which has evolutionarily acquired a phage-encoded OAg glucosylation, reported to have optimized the length of the OAg for T3SS function without compromising the protective properties of the LPS [24]. Shielding of T3SS by a G4C has also been found in EPEC and EHEC [31]. Still, expression of G4C has been shown to be required for efficient host colonization by EHEC *in vivo*, and to be inversely regulated to T3SS expression during different stages of pathogenesis [31]. Therefore, we used the rabbit model of experimental shigellosis to investigate G4C contribution to pathogenicity of *S*. *sonnei in vivo* and to the induction of inflammatory host responses during the infection. Analysis of infected rabbit tissues indicated that the lack of the capsule in *S*. *sonnei* Δ*g4c* caused a dramatic increase of pathogenicity and inflammatory potential in the gut, particularly characterized by the augmentation of mucus and blood production in the infected loop, rupture and destruction of the epithelial lining, and tissue inflammatory manifestations as determined by villi atrophy, submucosal edema, and Ameho grading. In addition, induction of cytokine production of the infected epithelium was higher in *S*. *sonnei* Δ*g4c*-infected loops than in *S*. *sonnei* WT-infected loops at the infectious dose of 5x10^9^ bacteria/loop. We hypothesize that in uncapsulated bacteria, virulence factors are not shielded by the capsule polysaccharide and this facilitates bacterial adhesion and entry in the host cells. Concomitantly, the recognition of invasive bacteria by the innate immune system of the host could be enhanced in the absence of the capsule, as demonstrated for *Neisseria* [46], further augmenting the inflammatory response. In contrast, hyper-encapsulated *S*. *sonnei* Δ*g4c*(*g4c*) strain showed an attenuated phenotype, with less bacteria invading the epithelium and less tissue inflammation compared to WT. We cannot exclude that in addition to the larger amount of capsule other factors contribute to the attenuated phenotype as we could not detect IpaB by Western blot; and it is not clear if this is a technical artefact due to interference of the capsule material with the immunodetection or a true lack of IpaB in *S*. *sonnei* Δ*g4c*(*g4c*). Moreover, the reduced pathogenicity of G4C-overexpressing *S*. *sonnei* Δ*g4c*(*g4c*) could be related not only to reduced invasiveness but also to higher host tolerance to encapsulated bacteria, as was shown for *Salmonella* serovars expressing Vi capsule [47].

In contrast to the stronger pathogenicity in the gut, lack of G4C reduced the ability of *S*. *sonnei* to spread to systemic infection sites. This was in accordance with enhanced complement sensitivity of *S*. *sonnei* Δ*g4c* compared to the WT. Similarly, the presence of the very long OAg was shown to be important for resistance to direct complement-mediated serum killing in *S*. *flexneri* 2a [48]. Complement resistance could play a role at the stage of inflammation in epithelial lesions involving complement recruitment [48] and at the stage of systemic disease which is increasing in children and immunocompromised patients [7,8]. Therefore, the capsule could be an important virulence factor for *S*. *sonnei* to survive host killing, both locally and systemically, and might play a similar role in infection as the very long OAg in *S*. *flexneri* 2a. Capsule deficiency could be beneficial in the early stages of the infection for proficiently invading the intestinal epithelium, but with a disadvantage in translocation. If G4C expression in *S*. *sonnei* is regulated during pathogenesis as the G4C in EHEC [31], or in a growth-dependent manner as the very long OAg in *S*. *flexneri* [22] remains to be addressed.

In conclusion, in *S*. *sonnei* expression of the capsule modulates virulence and results in a successful phenotype *in vivo*, with balanced capabilities to invade and persist in the host environment. Based on the substantial changes in pathogenesis observed upon deletion or overexpression of the *S*. *sonnei* capsule, we hypothesize that its level of expression may be under significant evolutionary pressure.

## Materials and Methods

### Bacterial strains, mutant construction, and growth conditions

*Shigella* strains used in this work are listed and described in Table 1. All *S*. *sonnei* strains are derivatives of *S*. *sonnei* 53G [49] (*S*. *sonnei* WT), all *S*. *flexneri* 2a strains are derivatives of *S*. *flexneri* 2a 2457T [50]. The *S*. *sonnei* Phase II colony (*S*. *sonnei* -pSS) was isolated during *in vitro* cultivation of *S*. *sonnei* WT bacteria on TSB Congo red agar plates by screening for spontaneous loss of pSS [33]. The experimentally induced *S*. *sonnei* -pSS nalidixic acid resistant (NA^R^) strain was isolated by serial passages of *S*. *sonnei* -pSS on LB agar plates supplemented with increasing concentrations of nalidixic acid (from 10 to 50 μg/mL). *S*. *sonnei* Δ*galU* was obtained using the same 3-step PCR method and materials as for generation of *S*. *sonnei* Δ*tolR*Δ*galU* [33]. OAg deletion in *S*. *flexneri* 2a was performed as previously described [51].

**Table 1:** *Shigella* strains used in this study, their abbreviation and a brief description of their phenotype.

Strain Nomenclature and Genetic Background	Description	Source
***S. sonnei* WT** *S. sonnei* 53G, +pSS	Phase I, carrying OAg-encoding virulence plasmid (pSS)	[49]
***S. sonnei* Δ*galU*** Δ*galU*::*cat*, +pSS	LPS deep rough mutant. Knockout of *galU* blocks the synthesis of uridine diphosphoglucose (UDP-glucose), resulting in an incomplete LPS core with no OAg attached	This study
***S. sonnei*—pSS** *S. sonnei* 53G, -pSS	Phase II, LPS OAg-deficient rough mutant. Avirulent	This study
***S. sonnei* -pSS NA** ^**R**^ *S. sonnei* 53G, -pSS	*S. sonnei* -pSS, resistant to nalidixic acid. Used in *in vivo* experiments	This study
***S. sonnei* ΔOAg** Δ*wbg*::*cat*, +pSS	LPS OAg-deficient rough mutant. Knockout of *wbg* OAg biosynthesis cluster (genes *wzz* to *wbgZ*) on pSS. Growth on Cm selects for the presence of pSS	This study
***S. sonnei* Δ*g4c*** Δ*g4c*::*erm*, +pSS	Group 4 capsule-deficient mutant. Knockout of *g4c* cluster coding sequence (genes *ymcD* to *etk*)	This study
***S. sonnei* Δ*g4c*(*g4c***) Δ*g4c*::*erm* +pACYC(*g4c*), +pSS	Complementation of Δ*g4c* null mutation using pACYC184 carrying functional *g4c* gene cluster. Growth on Cm selects for pACYC(*g4c*)	This study
***S. sonnei* Δ*virG*** Δ*virG*::*cat*, +pSS	Gene *virG* in pSS replaced by chloramphenicol resistance gene. Growth on Cm selects for the presence of pSS and thus avoids loss of the *wbg* OAg cluster *in vitro*. WT LPS and capsule. Attenuated	This study
***S. sonnei* Δ*tolR*** Δ*tolR*::*kan*, +pSS	Hyperblebbing *S. sonnei* WT. WT LPS and capsule	[33]
***S. sonnei* Δ*tolR*Δ*galU*** Δ*tolR*::*kan*, Δ*galU*::*cat*, +pSS	Hyperblebbing *S. sonnei* Δ*galU*. LPS deep rough mutant	[33]
***S. sonnei* Δ*tolR* -pSS** Δ*tolR*::*kan*, -pSS	Hyperblebbing *S. sonnei* -pSS. LPS OAg-deficient rough mutant	[33]
***S. sonnei* Δ*tolR*Δ*OAg*** Δ*tolR*::*kan*, Δ*wbg*::*cat*, +pSS	Hyperblebbing *S. sonnei* ΔOAg. LPS OAg-deficient rough mutant	This study
***S. sonnei* Δ*tolR*Δ*virG*** Δ*tolR*::*kan*, Δ*virG*::*cat*, +pSS	Hyperblebbing *S. sonnei* Δ*virG*. WT LPS and capsule	This study
***S. sonnei* Δ*tolR*Δ*virG*Δ*g4c*** Δ*tolR*::*kan*, Δ*virG*::*cat*, Δ*g4c*::*erm*,	Hyperblebbing *S. sonnei* Δ*g4c*, with stabilized pSS-driven OAg expression. WT LPS, capsule-deficient	This study
***S. flexneri* 2a WT** *S. flexneri* 2a 2457T, +pINV	WT LPS, carrying virulence plasmid (pINV)	[50]
***S. flexneri* 2a Δ*galU*** Δ*galU*::Tn*10*, +pINV	LPS deep rough mutant. Knockout of *galU* results in an incomplete LPS core with no OAg attached	[21]
***S. flexneri* 2a ΔOAg** Δ*rfbG*::*erm*, +pINV	LPS OAg-deficient rough mutant. Knockout of *rfbG* and partial deletion of flanking genes *rfbF* and *rfc* in OAg biosynthesis cluster on the chromosome	[51]

For generating the other knockout mutants, the chloramphenicol resistance gene (*cat*) from pKOBEG [52] was used to replace *S*. *sonnei virG* [53], *galU* [21], and the *wbg* cluster, encoding the biosynthesis of the O repeating units, from gene *wzz* to *wbgZ* on pSS [20]. Erythromycin resistance gene (*erm*) from pAT110 [54] was used to replace the *S*. *sonnei g4c* operon coding sequence, from gene *ymcD* to gene *etk* [30]. Upstream and downstream flanking regions of the locus to be deleted and the antibiotic resistance gene chosen for replacement were amplified using the primers described in Table 2 and inserted into pBluescript (Stratagene), so that the antibiotic resistance cassette interposed the flanking regions. A linear replacement construct (upstream region—resistance cassette—downstream region) was amplified by PCR and transformed into recombination-prone *Shigella* cells, produced by using the highly proficient homologous recombination system (red operon) [55] encoded on pAJD434 [56]. The deletion of the *g4c* operon in *S*. *sonnei* Δ*g4c* was complemented *in trans* as follows. The *g4c* gene cluster with the 280 bp upstream region was amplified by PCR (LongRange PCR Kit, QIAGEN) and cloned in pACYC184 (New England BioLabs) yield pACYC(*g4c*). *g4c* complementation in *S*. *sonnei* Δ*g4c*(*g4c*) was checked by exopolysaccharide expression.

**Table 2:** Primers used in this study.

DELETION	PRIMERS	SEQUENCE
ΔOAg (*S. sonnei*)	wzz-5 Sac	ACTCGAGCTCGGCAGACTCAGCGCAAG
	wzz-3 Sma	CTAACCCGGGCATTGACACAACAATACGTAACCCAG
	wgbZ-5 Sma	CTAACCCGGGTGCGATTTGGTAATGTACTCGG
	wgbZ-3 SalI	ACGCGTCGACATTGCTCGCTTGTGATAACAGC
Δ*g4c*	EcoRV.cps.3’.F	AGCTTGATATCcgcatggacaatacgtacgc
	XhoI.cps.3’.R	CCGCTCGAGacgtgtggaattttgccgcg
	XbaI.cps.5’.F	CTAGTCTAGAgatcaagacagcgttgacgc
	EcoRV.cps.5’.R	AGCTTGATATCaactgaaagtggccggatgc
Δ*virG*	virGup-5 Sac	ACTCGAGCTCTGTAGTTGATTTGACAGTTGACATCC
	virGup-3 Sma	CTAACCCGGGCACTATATTATCAGTAAGTGGTTGATAAACC
	virGdown-5 Sma	CTAACCCGGGCGTGTTGATGTCCTGC
	virGdown-3 Sal	ACGCGTCGACAGTTCAGTTCAGGCTGTACGC
INSERTION	PRIMER	SEQUENCE
*erm*	EcoRV.Ery.F	AGCTTGATATCAGAGTGTGTTGATAGTGCAGTATC
	EcoRV.Ery.R	AGCTTGATATCACCTCTTTAGCTTCTTGGAAGCT
*cat*	EcoRV.Cm.F	AGCTTGATATCTGTGACGGAAGATCACTTCG
	EcoRV.Cm.R	AGCTTGATATCGGGCACCAATAACTGCCTTA
*g4c* [pACYC(*g4c*)]	XbaI.COMPLcps.5’.F	CTAGTCTAGACATCCGGCCACTTTCAGTTTTAC
	SalI.COMPLcps.3’.R	ACGCGTCGACCAGCCAGTTATAGTACCACTTG

*E*. *coli* and *Shigella* strains were routinely cultured in LB or in TSB medium. When needed, growth media were supplemented with 30 μg/mL kanamycin, 20 μg/mL chloramphenicol, 100 μg/mL erythromycin, 50 μg/mL nalidixic acid, 100 μg/mL trimethoprim, 100 μg/mL ampicillin.

### GMMA preparation

GMMA were prepared as previously described [33] or from flask cultures as follows. Bacteria were grown in TSB medium in 1 L flasks to an OD_600_ of 5. Culture supernatants were collected by 10 min centrifugation at 4000 g and 0.22 μm filtered, concentrated using a 100 KDa regenerated cellulose membrane (Millipore) in a Stirred Ultrafiltration Cells (Amicon), separated from soluble proteins by 2 h ultracentrifugation at 186000 g at 4°C (Optima L‐series, 45Ti rotor, Beckman Instruments) and resuspended in phosphate buffer saline (PBS). All preparations were 0.22 μm filtered. GMMA protein concentration was quantified by Bradford Assay (Bio-Rad Protein Assay) using Bovine Serum Albumin (BSA, Thermo Scientific Pierce) as standard.

### Mice immunization

Groups of 8 CD1 female mice of 4 to 6 weeks of age were immunized subcutaneously on days 0, 21 and 35 with 2 μg of GMMA of *S*. *sonnei* Δ*tolR*, *S*. *sonnei* Δ*tolR*Δ*galU*, and *S*. *sonnei* Δ*tolR* -pSS strains in 100 μL PBS, or with PBS alone. Blood samples were collected before the first immunization (preimmune sera) and 14 days after the third injection.

### Bacterial surface staining coupled with flow cytometry analysis

Overnight (ON) *Shigella* cultures were diluted in TSB medium to OD_600_ = 0.05 and grown to OD_600_ = 0.5 (2.5x10^8^ CFU/mL). Cells were collected and diluted to 2x10^7^ CFU/mL. For formalin fixation, bacteria were diluted in 0.5% formalin solution in PBS and fixed with agitation ON at room temperature. Live or fixed bacteria were washed and resuspended in TSB. Primary staining was performed with mouse sera, the IpaB mouse monoclonal antibody (H16) [57], the *S*. *sonnei* Phase I monovalent rabbit antiserum (Denka Seiken, cat.# 295316), or the *S*. *flexneri* type II monovalent rabbit antiserum (Denka Seiken, cat.# 295019) at the desired dilution for 1 h at 4°C. For competitive staining experiments, 1:1000 diluted anti-*S*. *sonnei* Δ*galU* GMMA sera were incubated with 11.25 μg of LPS (as quantified by phenol-sulfuric assay) [58] extracted from *S*. *sonnei* WT or *S*. *sonnei* -pSS bacteria, for 1 h at 4°C in PBS, prior to bacterial staining. After washing with 1% BSA in PBS, bacteria were incubated with Allophycocyanin-conjugated AffiniPure F(ab')2 fragment Goat Anti-Mouse IgG (Jackson ImmunoResearch) or with Alexa Fluor 488 F(ab′)2 fragment of Goat Anti-Rabbit IgG (H+L) (Invitrogen) antibodies for 1 h on ice. Bacteria were washed, fixed with 4% formalin in PBS for 20 min on ice and analyzed for cell-bound fluorescence using a FACSCanto II flow cytometer (BD Biosciences). To evaluate IpaB exposure on the surface of *S*. *sonnei* WT, *S*. *sonnei* Δ*g4c*, *S*. *sonnei* Δ*g4c*(*g4c*), *S*. *sonnei* ΔOAg and *S*. *sonnei* -pSS bacteria were stained with the 1:50 dilution of the IpaB monoclonal antibody. For quantitative analyses, the differential Mean Fluorescence Intensity (ΔMFI) was measured as the difference between the MFI of the immune staining and the MFI of the control staining using the secondary antibody alone. Flow cytometry data were processed using FlowJo software (Tree Star).

### LPS and capsule isolation

LPS and G4C crude extracts were prepared by the phenol-water method [59], with modifications. For total cellular extraction, ON *Shigella* cultures were diluted in 50 mL LB supplemented with antibiotics, if needed, to OD_600_ = 0.1 and grown until OD_600_ = 1. Bacteria were collected by centrifugation and resuspended in 500 μL PBS. After 5 min boiling, the mixture was treated with 0.5 μg/μL of Proteinase K (Thermo Scientific Pierce) at 60°C ON. An equal volume of saturated phenol solution (pH 8.0) (Sigma-Aldrich) was added and incubated for 1 h at 70°C with occasional mixing. After 1 h centrifugation at 10000 g, the upper aqueous phase was mixed with 2 volumes of absolute ethanol and incubated for 1 h at -70°C. Samples were centrifuged at 12000 g for 30 min and the pellet containing LPS and capsule polysaccharides was dried in a rotary vacuum drier (SpeedVac) and dissolved in distilled water. To obtain LPS and capsule material with minimal protein and nucleic acids contaminants, GMMA were used as starting outer membrane preparations. After Proteinase K incubation step, samples were treated as described above. The lipid A moiety of LPS was subsequently removed from phenol-water extracts by mild acid hydrolysis treatment with 1% acetic acid for 1.5 h at 100°C and adjusted to pH 6.5 with ammonium hydroxide. Samples were centrifuged at 15000 g ON and the supernatant, containing exopolysaccharide, was collected and dialyzed against distilled water.

### Silver stained SDS-PAGE and western blot

Phenol-water extracts and total cell lysates were analyzed by 12% Bis‐Tris Sodium Dodecyl Sulfate Polyacrylamide Gel Electrophoresis (SDS-PAGE) and silver stained using the SilverQuest Silver Staining Kit (Invitrogen). Samples were transferred to nitrocellulose membranes and incubated with a 1:1000 dilution of the primary antibody, washed and then incubated with a 1:5000 dilution of the appropriate secondary antibody (Goat anti-Mouse IgG-Alkaline Phosphatase antibody, Sigma-Aldrich, or Goat anti-Rabbit IgG-Alkaline Phosphatase conjugate, Invitrogen). Immunoblots were developed using the SIGMAFAST BCIP/NBT tablet (Sigma-Aldrich) solution. At least two independent experiments were performed. Representative blots are shown.

### Polysaccharide molecular weight distribution analysis

High Performance Liquid Chromatography-Size Exclusion Chromatography (HPLC-SEC) analysis was used to analyze the size distribution of exopolysaccharide populations and to isolate fractions of different molecular weight. Acid-cleaved exopolysaccharide samples were run on a TSK gel G3000 PWXL column (30 cm X 7.8 mm; particle size 7 μm; cat.# 808021) with a TSK gel PWXL guard column (4.0 cm X 6.0 mm; particle size 12 μm; cat.# 808033) (Tosoh Bioscience). The mobile phase was 0.1 M NaCl, 0.1 M NaH_2_PO_4_, 5% CH_3_CN, pH 7.2 at a flow rate of 0.5 mL/min (isocratic method for 30 min). Void and bed volumes were calibrated with λ-DNA (λ-DNA Molecular Weight Marker III 0.12–21.2 Kbp, Roche) and sodium azide (Merck), respectively. Column void volume (T_0_): 10.58 min; total volume (T_tot_): 23.03 min. Distribution coefficient, Kd = (T_retention_—T_0_)/(T_tot—_T_0_). Polysaccharide peaks were detected by differential refractive index (dRI) or at 214 nm, when run with a dextran standard curve (270–12 kDa range) to calculate the apparent average molecular weight. Exopolysaccharide was fractionated and collected as follow: HMW-PS, from 11.5 min to 13 min; MMW-PS, from 14 min to 16 min; LMW-PS, from 17 min to 18 min.

### H^1^-NMR

1D proton NMR analysis was performed to confirm the identity of the polysaccharide samples. NMR spectra of isolated Phase I exopolysaccharide fractions were collected using a standard one-pulse experiment after solubilization of polysaccharide samples in deuterated water. Experiments were recorded at 25°C on Varian VNMRS-500 spectrometer, equipped with a Pentaprobe. Chemical shifts were referenced to hydrogen deuterium oxide (HDO) at 4.79 ppm. For data acquisition and processing VNMRJ ver. 2.2 rev. C and Mestrenova 6.1 (Mestrelab Research) were used respectively.

### Electron microscopy of bacterial ultrathin sections

For alcian blue staining, single colonies were looped directly from the plate into fixative containing 2.5% glutaraldehyde, 2% paraformaldehyde, 0.075% alcian blue 8 GX and 50 mM L-lysine monohydrochloride in 0.025 M sodium acetate buffer at pH 5.7 for 2 hours at room temperature. Samples were then rinsed 3 times in buffer and post-fixed in 1% osmium tetroxide in 0.1 M sodium cacodylate buffer, rinsed again, dehydrated through an ethanol series, en bloc stained with 2% uranyl acetate at the 30% stage and embedded in TAAB 812 resin. Ultrathin 60 nm sections were cut on a Leica EM UC6 ultramicrotome, contrasted with uranyl acetate and lead citrate, examined on a 120 kV FEI Spirit Biotwin and imaged with a Tietz F4.15 CCD camera.

For immunogold-labelling, single colonies were looped into 2% paraformaldehyde and 0.25% glutaraldehyde in 0.1 M phosphate buffer (PB) at pH 7.4 for 1 hour at room temperature, rinsed 3 times in buffer, infiltrated with 1% and then 10% gelatin before immersing in 2.3 M sucrose in PB ON at 4°C for cryoprotection. Frozen samples were prepared by mounting onto aluminum pins and rapidly immersing in liquid nitrogen in preparation for ultrathin 80 nm sectioning on a Leica EM FC6 ultramicrotome. Ultrathin sections were labelled as per Tokuyasu [60], with the *S*. *sonnei* Phase I monovalent rabbit antiserum (dil. 1:25) and detected with 10 nm protein A gold. Imaging was performed as above. Thickness of Phase I material extending beyond the outer membrane was evaluated on different micrographs fields, as average of 20 measurements along the bacterial surface.

### HeLa cells invasion assay

Invasiveness of the different *S*. *sonnei* strains was evaluated *in vitro* by using a gentamicin protection assay conducted on HeLa semiconfluent monolayers. Each condition was tested in triplicate in three independent experiments. Briefly, 5x10^5^ HeLa cells/well (HeLa ATCC, CCL-2) were seeded ON in 6 well cell culture plates (Corning Costar) in Dulbecco’s Modified Eagle Medium (DMEM) high Glucose (Invitrogen), supplemented with 10% FBS (New Zealand, Invitrogen). The following day, *Shigella* ON cultures were diluted in TSB medium (supplemented with antibiotics, if needed) and grown to OD_600_ = 0.5. Bacteria were collected, diluted in DMEM and used to infect HeLa cells with a Multiplicity of Infection (MOI) of 10 bacteria/cell. After the addition of the bacteria, the cells were centrifuged at 1100 g for 18 min at 37°C and incubated for 1 h at 37°C. Subsequently, the monolayers were washed with PBS and the medium was replaced with DMEM containing 80 μg/mL gentamicin, to kill extracellular bacteria. After 2 h cells were washed and lysed by the addition of cold 0.5% sodium deoxycholate (Sigma-Aldrich) in water. Suitable dilutions were plated in triplicates on Congo red agar plates to determine the number of recovered viable intracellular bacteria.

### Complement sensitivity assay

*Shigella* ON cultures were diluted in TSB medium and grown to OD_600_ = 0.5, equivalent to 2.5x10^8^ *Shigella* bacteria/mL. Bacteria were collected, diluted to 10,000/mL in PBS and used as 10x suspension for the assay. Lyophilized baby rabbit complement (Cedarlane, CL3441) was reconstituted with 1 mL of sterile MilliQ water. The bacteria were mixed with reconstituted complement and PBS to yield 50%, 75%, 90% final complement concentration and an inoculum of 1000 bacteria/mL. Heat-inactivated complement was used as control. Bacterial counts were determined by plating on Congo Rod agar at time zero and after 3 h incubation at 37°C. To determine complement sensitivity of *S*. *sonnei* WT and *S*. *sonnei* Δ*g4c* only red colonies possessing the OAg-encoding pSS virulence plasmid were counted at time zero as white colonies lacking pSS and thus the OAg are highly complement sensitive [38]. The results are expressed as x-fold increase/decrease of the cell count after incubation compared to the count at time zero (inoculum).

### Rabbit ligated ileal loop model

Virulence of the different *S*. *sonnei* strains was evaluated *in vivo* by testing their ability to induce a *Shigella*-dependent gut pathology and to spread to systemic sites in the rabbit model of ligated ileal loops in New Zealand White rabbits weighing 2.5–3 kg (Charles River Breeding Laboratories, Wilmington, MA). In the main experiment, each loop was infected with 3x10^9^ bacteria of a single strain in 500 μL of physiological saline buffer (0.9% NaCl). 12 loops per animal of 5 cm segments of ileum starting at the ileum-cecum transition were ligated, avoiding all Peyer’s patches, while maintaining the existing vasculature. Each strain was tested in 2 rabbits, in 3 replicate loops per rabbit, with a randomized order for each rabbit. Surgery was performed as described [61]. In a preliminary experiment, 5x10^9^ bacteria/loop infectious dose was tested in 2 loops for each strain in 2 rabbits. After euthanasia, loops were dissected and processed for RNA extraction and histology. Uninfected tissues were collected as control. Mesenteric lymph nodes, spleen, liver and blood were collected and processed for bacterial counting. Appropriate dilutions of the different tissue samples were plated on selective and non-selective TSB-agar: *S*. *sonnei* Δ*g4c*(*g4c*) was identified by growth on chloramphenicol; *S*. *sonnei* Δ*g4c* was enumerated by growth on erythromycin minus the counts of *S*. *sonnei* Δ*g4c*(*g4c*) determined on chloramphenicol as *S*. *sonnei* Δ*g4c*(*g4c*) is resistant to both; *S*. *sonnei* -pSS was selected on nalidixic acid; *S*. *sonnei* WT does not carry any resistance markers and thus was counted by plating on non-selective medium and subtracting the number of the other strains determined on the selective media from the total number of CFU obtained on non-selective medium.

### Tissue pathology analysis

For histopathological analysis of *Shigella* infected ileal loops and bacterial immune-localization, intestinal biopsies were fixed at 4°C in 4% paraformaldehyde in PBS, embedded in paraffin and sectioned into 7 μm slices using a microtome. Sections were deparaffinated, rehydrated and used for H&E staining, or for anti-*Shigella* immune staining.

Immune staining was performed as follows. Sections were permeabilized for 15 min with antigen unmasking solution (10 mM Tris, 1 mM EDTA, 0.05% Tween20, pH 9), treated with 3.3% H_2_O_2_ for 10 min and washed. Samples were blocked for 15 min with Ultra V block (Lab Vision Corp; Thermo Scientific, cat.# TA-125UB) and incubated ON with the in-house mouse polyclonal anti-*S*. *sonnei* serum raised against *S*. *sonnei* Δ*tolR* GMMA. Samples were then incubated with a peroxidase labelled polymer conjugated to goat anti-mouse immunoglobulins (DAKO, cat.# K4000) for 1 h, revealed with the 3-amino-9-ethylcarbzole AEC+ Substrate-Chromogen (DAKO, cat.# K3461), counterstained with hematoxylin and mounted with aqueous mounting medium (Merck). Histology images were taken using light microscopy, at 4X magnification for H&E staining, and at 20X magnification for immune staining.

*Shigella*-dependent intestinal inflammation and the degree of tissue alteration were assessed by evaluating the extent of villi atrophy and submucosal edema and by determining scores of inflammation according to the generally used criteria of the Ameho gradation scale [37], in slices from rabbit loops after 8 hours infection with 3x10^9^ bacteria/loop of a single strain.

Pro-inflammatory cytokines (IL-8, IL-6, and IL-1β) gene expression was measured by RT-qPCR in rabbit loops after 8 hours infection with 5x10^9^ bacteria/loop of a single strain. Primers and methods were previously described [61]. The expression levels of the cytokine genes were determined as fold induction over the housekeeping gene GAPDH in each loop. To compare expression levels elicited by *S*. *sonnei* Δ*g4c* and *S*. *sonnei* WT the ratio of the fold induction (over GAPDH) in *S*. *sonnei* Δ*g4c* infected loops and the fold induction in the adjacent *S*. *sonnei* WT infected loop was calculated.

### List of Gene ID numbers

*S*. *sonnei* genes (*S*. *sonnei* 046): *tolR*: 3669577; *virG*: 3670887; *galU*: 3667724; *wzz*: 3670967; *wzx*: 3670970; *wzy*: 3670971; *wbgZ*: 3670977; *ymcD*: 3666464; *ymcC*: 3666463; *ymcB*: 3666462; *ymcA*: 3666461; *yccZ*: 3669961; *etp* (*yccY*): 3669960; *etk (yccC)*: 3669959. *S*. *flexneri* genes (*S*. *flexneri* 2a 2457T): *tolR*: 1077009; *galU*: 1077674; *rfbG*: 1078499; *rfbF*: 1078523; *rfc*: 1078521.

### Ethics statement

The mouse immunization experiments performed at the Novartis Animal Facility in Siena, Italy, complied with the relevant guidelines of Italy (Italian Legislative Decree n. 116/1992) and the institutional policies of Novartis. The animal protocol was approved by the Animal Welfare Body of Novartis Vaccines, Siena, Italy, and by the Italian Ministry of Health (Approval number AEC 2009–05). The experiments using the rabbit ileal loop model performed at Institut Pasteur, Paris, France, complied with the EU Directive 2010/63 and the French Decree 2013–118. The respective protocol was approved by the Comité Regional d’Éthique pour l’Expérimentation Animale in Paris 1 (protocol no.20070004) and reviewed by the Global Animal Welfare Board of Novartis.

## Supporting Information

S1 Fig Deep rough *S*. *sonnei* Δ*galU* but not *S*. *flexneri* 2a Δ*galU* induce OAg-specific antibodies. Flow cytometry analysis of surface staining of live *S*. *sonnei* WT (left column) and *S*. *flexneri* 2a WT (right column) with mouse sera raised against 10^9^ formalin-fixed CFU of the corresponding WT (anti-WT), ΔOAg mutant with rough LPS (anti-ΔOAg), and Δ*galU* mutant with deep rough LPS (anti-Δ*galU*). Left column: *S*. *sonnei* WT was stained with sera raised against *S*. *sonnei* WT, *S*. *sonnei* ΔOAg generated by deletion of the *S*. *sonnei wgb* OAg biosynthesis cluster encoded on the virulence plasmid, or *S*. *sonnei* Δ*galU*. Right column: *S*. *flexneri* 2a WT (*S*. *flexneri* WT) was stained with sera raised against *S*. *flexneri* 2a WT, *S*. *flexneri* 2a ΔOAg generated by deletion of the *S*. *flexneri* 2a *rfb* OAg biosynthesis cluster encoded on the chromosome, or *S*. *flexneri* 2a Δ*galU*. Gray profiles: staining with preimmune sera; black profiles: staining with formalin-fixed bacteria antisera (*S*. *sonnei* (Ss) antisera binding and *S*. *flexneri* 2a (Sf) antisera binding). Sera dilution was 1:500. Representative results of three experiments are shown.
(TIFF)

S2 Fig *E*. *coli g4c* cluster is conserved in *S*. *sonnei* but not in *S*. *flexneri* 2a. Schematic representation of the *S*. *sonnei g4c* gene cluster (*ymcDCBA*, *yccZ*, *etp*, *etk*) in its genomic context with flanking genes (*cspH*, *appA*), built utilizing the Artemis browser (https://www.sanger.ac.uk/resources/software/artemis/) and the *S*. *sonnei* 046 genome sequence (NC_007384.1). In the *S*. *sonnei* Δ*g4c* mutant the complete cluster from the start of *ymcD* to the end of *etk* has been removed. An alignment of Etk amino acid sequences of *E*. *coli* (Ec) 0127:H6 E2348/69, *S*. *sonnei* (Ss) 046, Ss 53G, *S*. *flexneri* (Sf) 2a 2457T and Sf 2a 301 is shown. A deletion of 14 bases (from position 135 to 148) in the *etk* locus causes a frame-shift mutation (effective at amino acid K47) affecting Sf 2a 2457T and Sf 2a 301. Asterisks indicate the presence of a stop codon in the corresponding DNA sequence.
(TIFF)

S3 Fig High molecular weight polysaccharides are present in both *S*. *sonnei* Δ*galU* and Phase I exopolysaccharide. HPLC-SEC (dRI) analysis of acid-cleaved exopolysaccharide (EPS) purified from GMMA of hyperblebbing *S*. *sonnei* Δ*galU* (Δ*galU* EPS, dashed-dotted line) without stabilized virulence plasmid-driven expression of the OAg, in comparison to the trimodal Phase I EPS from *S*. *sonnei* GMMA (solid line). High molecular weight polysaccharides (HMW-PS) are present at similar retention time in both Δ*galU* and Phase I EPS. Samples were run on TosoHaas TSK gel G3000 PWXL-CP column.
(TIFF)

S4 Fig Acid-cleaved G4C polysaccharide does not contain the LPS core reducing sugar 3-deoxy-D-manno-octulosonic acid. Presence of 3-deoxy-D-manno-octulosonic acid (KDO) at the reducing end of isolated *S*. *sonnei* Phase I polysaccharide populations after acid cleavage was estimated by semicarbazide assay for α-ketoacids determination by HPLC-SEC analysis, as described [62] (reaction in the square box). Derivatized polysaccharide fractions (high, medium, low molecular weight polysaccharides, respectively HMW, MMW, LMW-PS) were run on TosoHaas TSK gel G3000 PWXL-CP column. Detection was at 252 nm. Solid line: HMW-PS; dashed line: MMW-PS; dotted line: LMW-PS.
(TIFF)

S5 Fig IpaB expression in *S*. *sonnei* strains. IpaB-specific immunoblot analysis of total cell lysates of *S*. *sonnei* WT, *S*. *sonnei* Δ*g4c*, *S*. *sonnei* ΔOAg and *S*. *sonnei* -pSS. 10^8^ bacteria/lane were run on 12% Bis-Tris SDS-PAGE, blotted, and membranes were incubated with the IpaB monoclonal antibody (anti-IpaB) at a dilution of 1:1000.
(TIFF)

S6 Fig Comparison of *S*. *sonnei* and *S*. *flexneri* 2a complement sensitivity. Sensitivity of *S*. *sonnei* WT (Ss WT) and *S*. *flexneri* 2a WT (Sf WT) to increasing concentrations of baby rabbit complement (50, 75, and 90%) in 3 h incubation. Assays were performed in triplicate in three independent experiments and the results are expressed as x-fold increase/decrease compared to the number of the bacteria in the inoculum (**p = 0.0087).
(TIF)

S1 Table Phase I-specific surface staining of live and formalin-fixed *S*. *sonnei* strains. Average values and standard deviations (SD) of the differential Mean Fluorescence Intensity (ΔMFI) of three independent surface staining experiments of live or formalin-fixed *S*. *sonnei* strains with *S*. *sonnei* Phase I monovalent antiserum (anti-Ss Phase I).
(PDF)

S2 Table *S*. *sonnei* 53G and 25931 isolates have exopolysaccharides with trimodal molecular weight distribution. Distribution coefficients (Kd) of high, medium and low molecular weight polysaccharides (HMW, MMW and LMW-PS) of acid-cleaved exopolysaccharide (EPS) purified from *S*. *sonnei* 53G and *S*. *sonnei* 25931 bacteria and analyzed by HPLC-SEC (dRI).
(PDF)
