# Supplementary figures and images for: An O Antigen Capsule Modulates Bacterial Pathogenesis in Shigella sonnei
Source: PLoS Pathog. 2015 Mar 20;11(3):e1004749. doi: 10.1371/journal.ppat.1004749 (PMC4368438; doi:10.1371/journal.ppat.1004749)

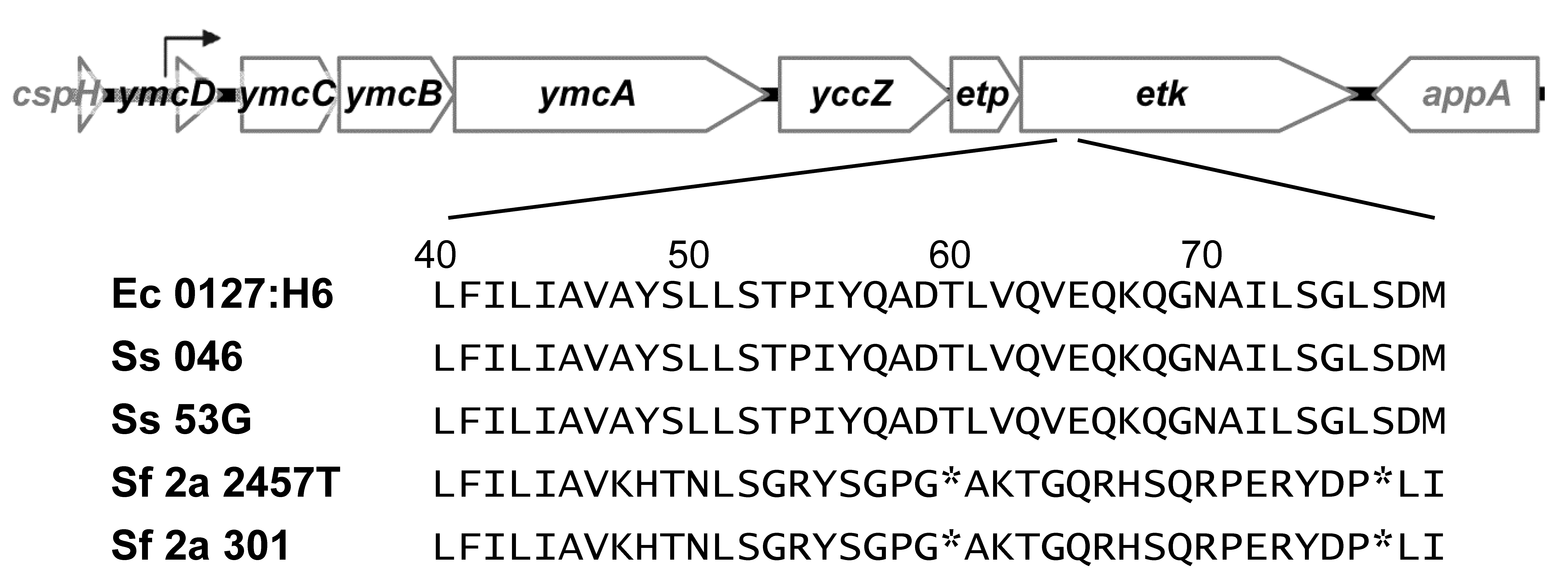

Supplement: S2 Fig — Schematic representation of the S. sonnei g4c gene cluster (ymcDCBA, yccZ, etp, etk) in its genomic context with flanking genes (cspH, appA), built utilizing the Artemis browser (https://www.sanger.ac.uk/resources/software/artemis/) and the S. sonnei 046 genome sequence (NC_007384.1). In the S. sonnei Δg4c mutant the complete cluster from the start of ymcD to the end of etk has been removed. An alignment of Etk amino acid sequences of E. coli (Ec) 0127:H6 E2348/69, S. sonnei (Ss) 046, Ss 53G, S. flexneri (Sf) 2a 2457T and Sf 2a 301 is shown. A deletion of 14 bases (from position 135 to 148) in the etk locus causes a frame-shift mutation (effective at amino acid K47) affecting Sf 2a 2457T and Sf 2a 301. Asterisks indicate the presence of a stop codon in the corresponding DNA sequence. (TIFF) [file ppat.1004749.s002.tiff]

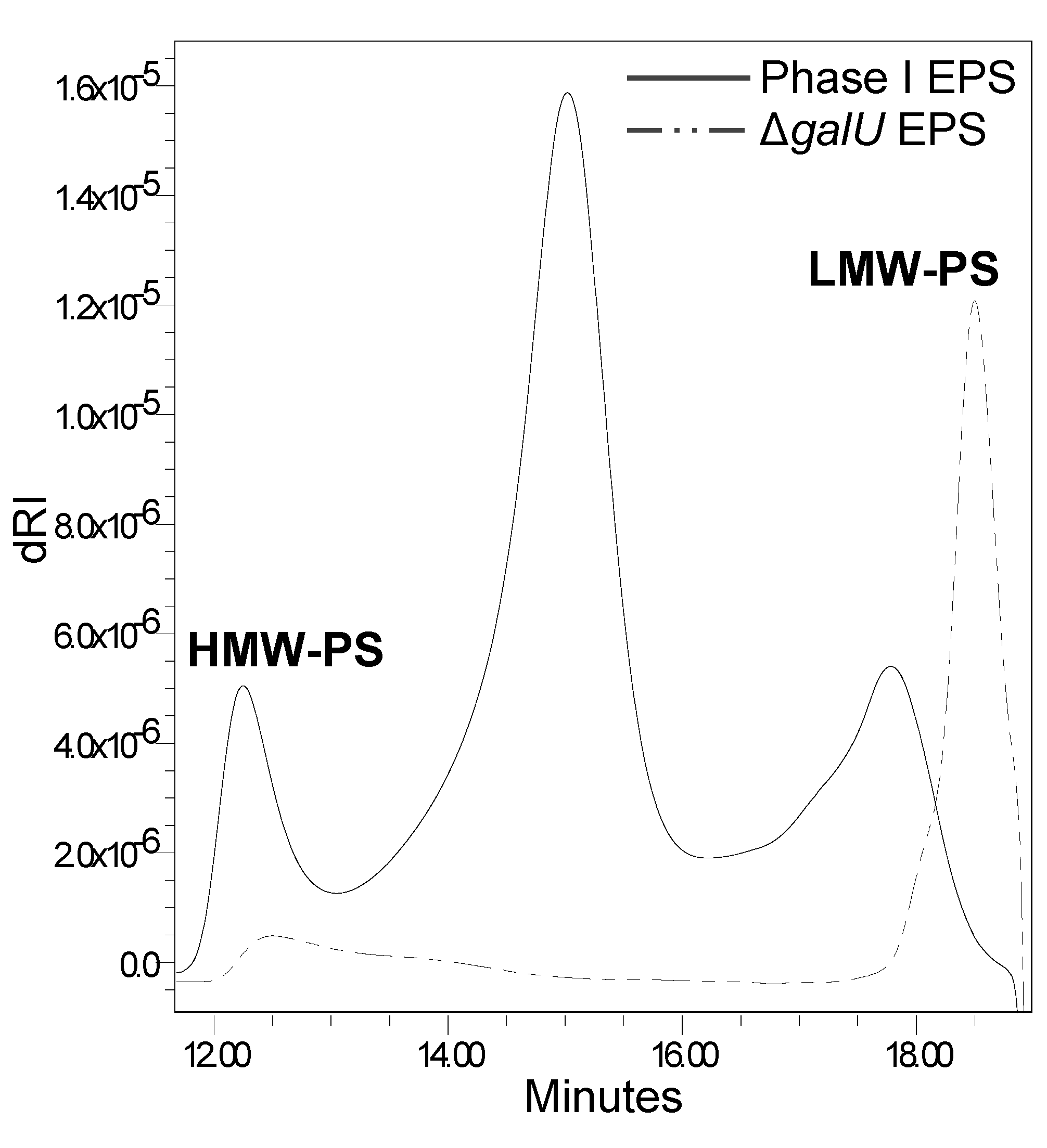

Supplement: S3 Fig — HPLC-SEC (dRI) analysis of acid-cleaved exopolysaccharide (EPS) purified from GMMA of hyperblebbing S. sonnei ΔgalU (ΔgalU EPS, dashed-dotted line) without stabilized virulence plasmid-driven expression of the OAg, in comparison to the trimodal Phase I EPS from S. sonnei GMMA (solid line). High molecular weight polysaccharides (HMW-PS) are present at similar retention time in both ΔgalU and Phase I EPS. Samples were run on TosoHaas TSK gel G3000 PWXL-CP column. (TIFF) [file ppat.1004749.s003.tiff]

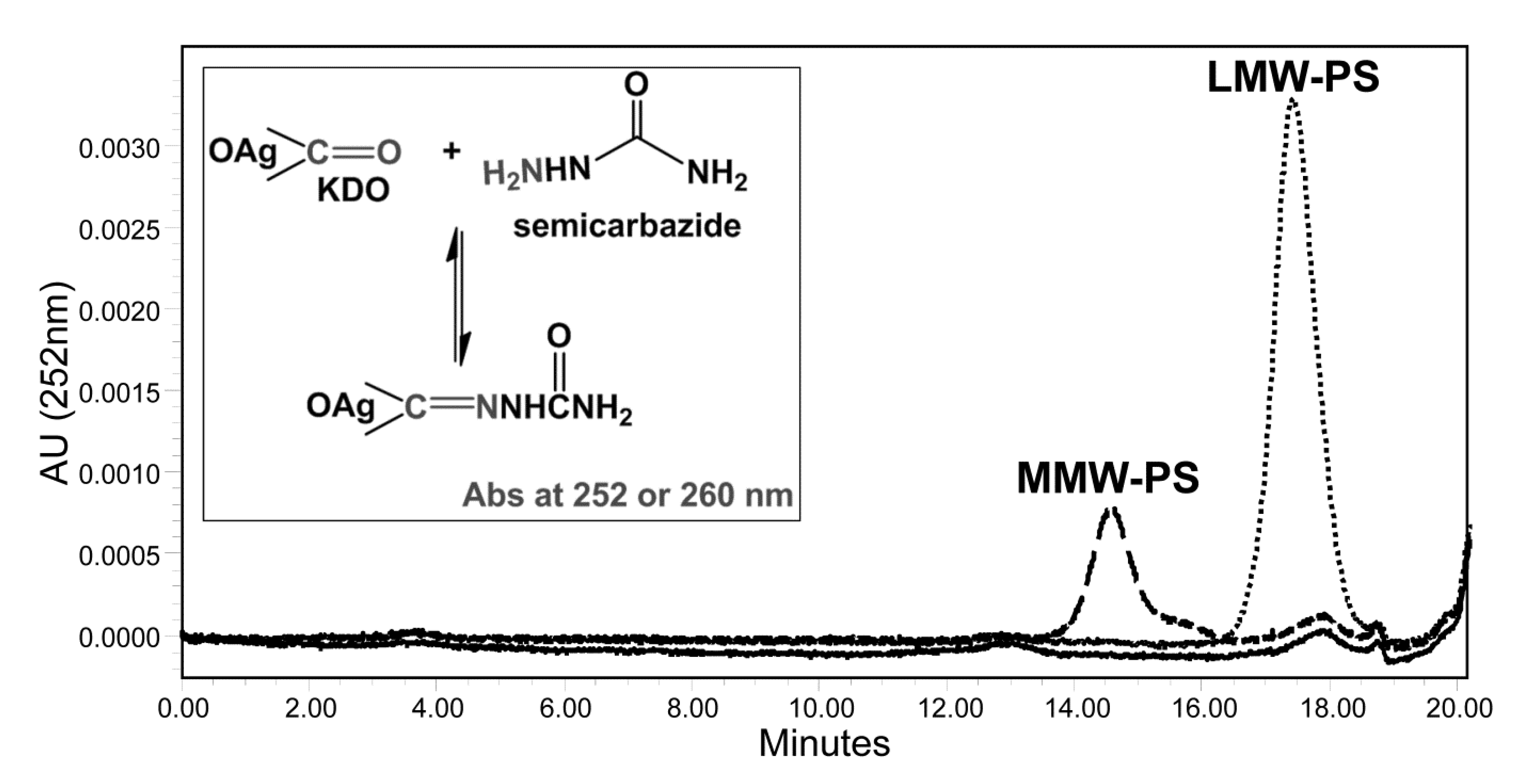

Supplement: S4 Fig — Presence of 3-deoxy-D-manno-octulosonic acid (KDO) at the reducing end of isolated S. sonnei Phase I polysaccharide populations after acid cleavage was estimated by semicarbazide assay for α-ketoacids determination by HPLC-SEC analysis, as described [62] (reaction in the square box). Derivatized polysaccharide fractions (high, medium, low molecular weight polysaccharides, respectively HMW, MMW, LMW-PS) were run on TosoHaas TSK gel G3000 PWXL-CP column. Detection was at 252 nm. Solid line: HMW-PS; dashed line: MMW-PS; dotted line: LMW-PS. (TIFF) [file ppat.1004749.s004.tiff]

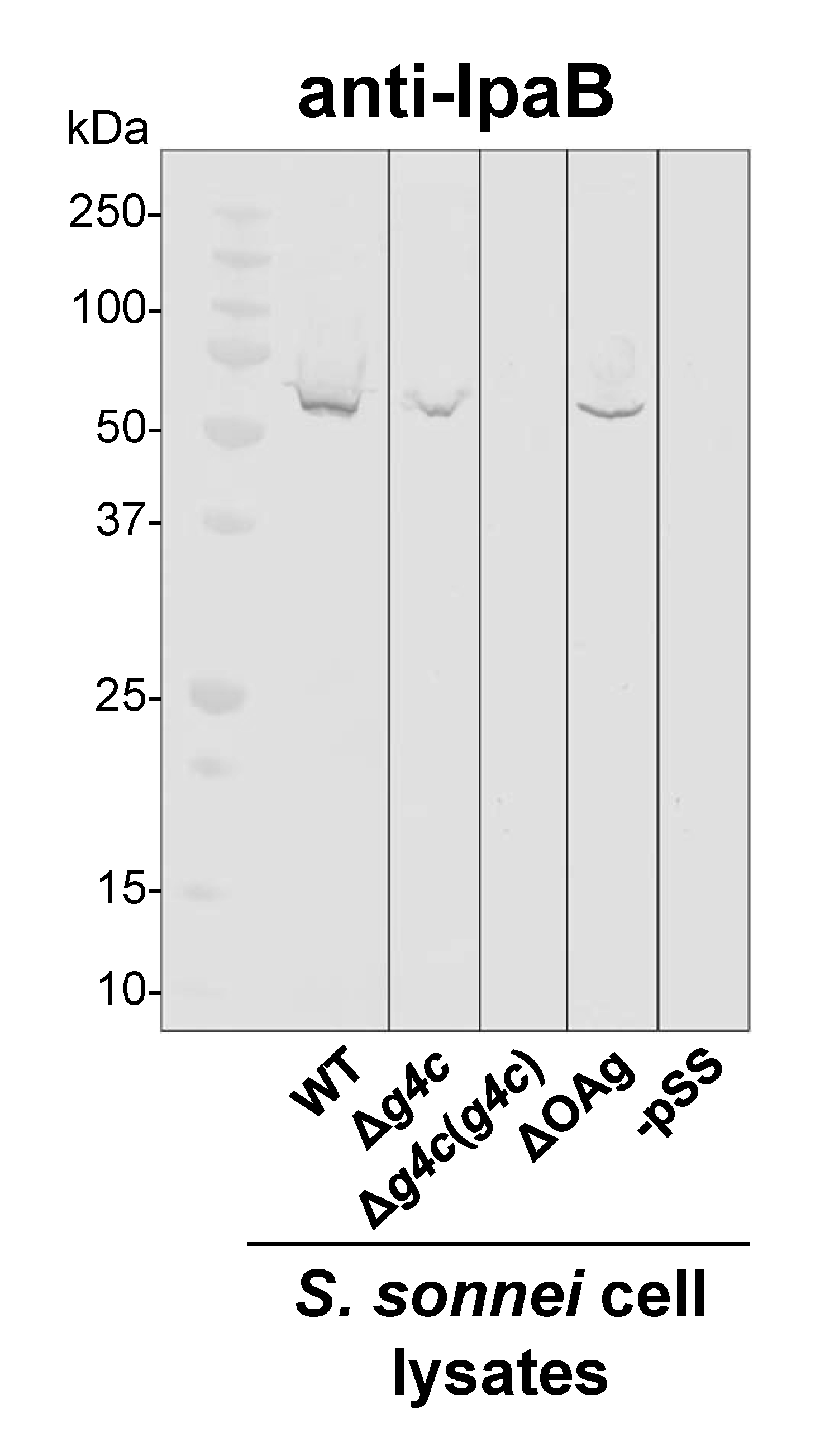

Supplement: S5 Fig — IpaB-specific immunoblot analysis of total cell lysates of S. sonnei WT, S. sonnei Δg4c, S. sonnei ΔOAg and S. sonnei -pSS. 108 bacteria/lane were run on 12% Bis-Tris SDS-PAGE, blotted, and membranes were incubated with the IpaB monoclonal antibody (anti-IpaB) at a dilution of 1:1000. (TIFF) [file ppat.1004749.s005.tiff]

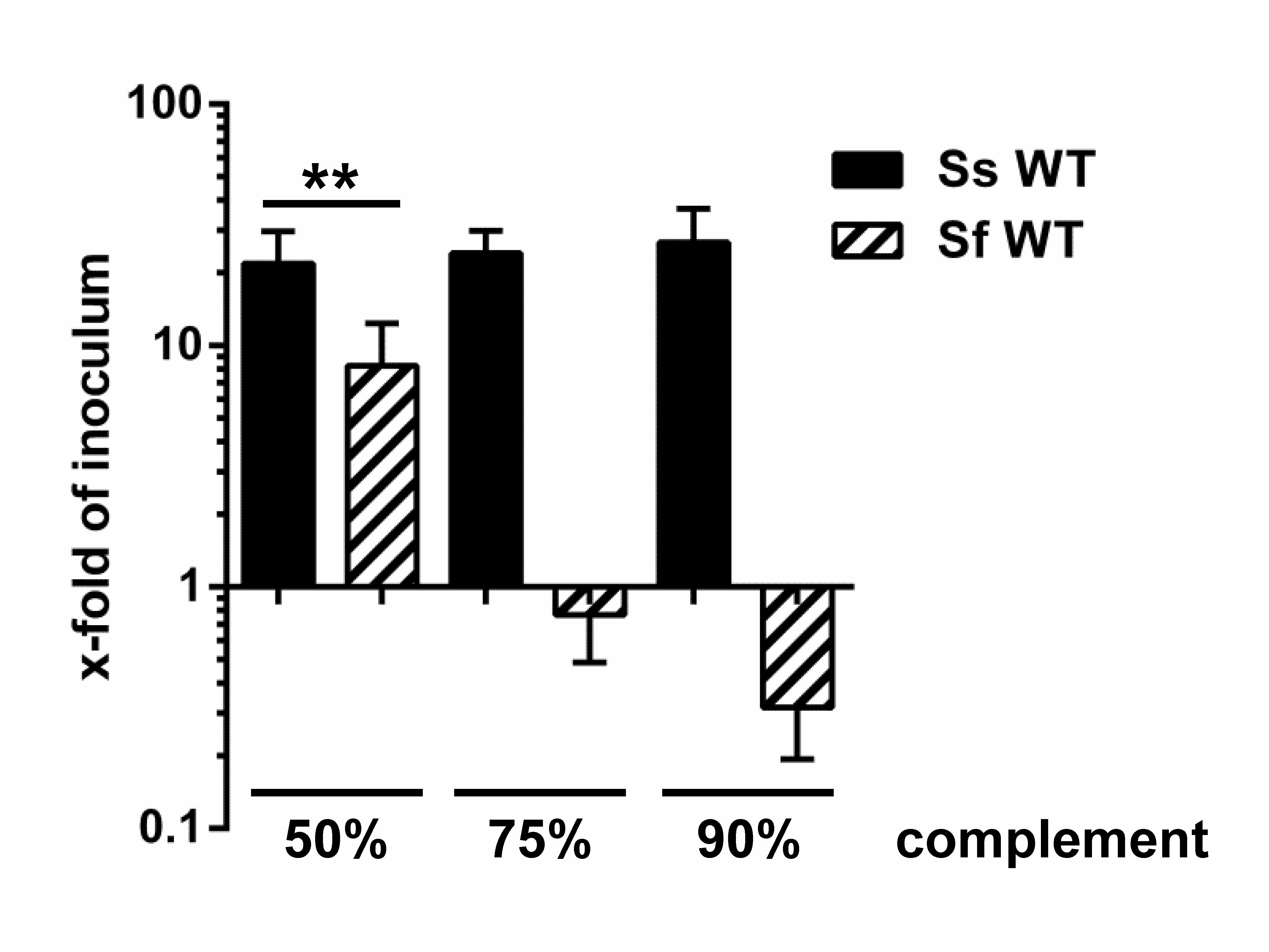

Supplement: S6 Fig — Sensitivity of S. sonnei WT (Ss WT) and S. flexneri 2a WT (Sf WT) to increasing concentrations of baby rabbit complement (50, 75, and 90%) in 3 h incubation. Assays were performed in triplicate in three independent experiments and the results are expressed as x-fold increase/decrease compared to the number of the bacteria in the inoculum (**p = 0.0087). (TIF) [file ppat.1004749.s006.tif]
