# Supplementary material for: An O Antigen Capsule Modulates Bacterial Pathogenesis in Shigella sonnei
Source: PLoS Pathog. 2015 Mar 20;11(3):e1004749. doi: 10.1371/journal.ppat.1004749 (PMC4368438; doi:10.1371/journal.ppat.1004749)
Supplement: S1 Table — Average values and standard deviations (SD) of the differential Mean Fluorescence Intensity (ΔMFI) of three independent surface staining experiments of live or formalin-fixed S. sonnei strains with S. sonnei Phase I monovalent antiserum (anti-Ss Phase I). (PDF) [file ppat.1004749.s007.pdf]

Strain	**anti-Ss Phase I ΔMFI** (mean±SD)	
	**Live**	**Formalin-fixed**
S. sonnei WT	14426 ± 2858	18136 ± 5033
S. sonnei Δ*galU*	623 ± 433	2503 ± 88
S. sonnei -pSS	19 ± 36	12 ± 17
