# Supplementary material for: An O Antigen Capsule Modulates Bacterial Pathogenesis in Shigella sonnei
Source: PLoS Pathog. 2015 Mar 20;11(3):e1004749. doi: 10.1371/journal.ppat.1004749 (PMC4368438; doi:10.1371/journal.ppat.1004749)
Supplement: S2 Table — Distribution coefficients (Kd) of high, medium and low molecular weight polysaccharides (HMW, MMW and LMW-PS) of acid-cleaved exopolysaccharide (EPS) purified from S. sonnei 53G and S. sonnei 25931 bacteria and analyzed by HPLC-SEC (dRI). (PDF) [file ppat.1004749.s008.pdf]

EPS populations (Kd)	*S. sonnei* 53G	*S. sonnei* 25931
HMW-PS	0.15	0.15
MMW-PS	0.45	0.42
LMW-PS	0.62	0.62
